# scCCVGBen for benchmarking of single-cell representation learning anchored on a centroid-coupled variational graph attention autoencoder across scRNA-seq and scATAC-seq

**DOI:** 10.3389/fgene.2026.1822168

**Published:** 2026-06-16

**Authors:** Zeyu Fu, Jiawei Fu, Chunlin Chen, Keyang Zhang, Junping Wang, Tianfei Ran, Song Wang

**Affiliations:** 1 State Key Laboratory of Trauma and Chemical Poisoning, Institute of Combined Injury, Chongqing Engineering Research Center for Nanomedicine, College of Preventive Medicine, Army Medical University, Chongqing, China; 2 Department of Orthopedics, Xinqiao Hospital, Army Medical University, Chongqing, China; 3 Department of Rehabilitation Medicine, The First Affiliated Hospital, Sun Yat-sen University, Guangzhou, China; 4 School of Medicine, Sun Yat-sen University, Shenzhen, China

**Keywords:** benchmarking, graph neural network, manifold learning, representation learning, representation stability, single-cell, variational autoencoder

## Abstract

Single-cell omics routinely profile millions of cells across the transcriptome and the epigenome. However, embeddings used for clustering, trajectory inference, and visualization remain unstable: stochastic variational autoencoders inject sampling noise at inference, and methods reported on idiosyncratic cohorts defeat head-to-head comparison. We introduce scCCVGBen, a benchmark of single-cell representation-learning methods. Its reference configuration is a centroid-coupled variational graph autoencoder built from three design choices: the centroid (deterministic posterior mean) used as the inference embedding, a coupling-regularized dual-reconstruction bottleneck, and a graph attention encoder over a k -nearest-neighbor cell–cell graph. We assess this configuration within a decoupled benchmark that varies the algorithmic core, encoder backbone, graph construction, dataset cohort, and evaluation suite as independent axes. The cohort, drawn from the Gene Expression Omnibus (GEO) and the European Nucleotide Archive (ENA), balances scRNA-seq and scATAC-seq equally and spans hematopoiesis, neuronal differentiation, immune populations, organ atlases, tumor microenvironments, and developmental time courses. Across the cohort, scCCVGBen improves average silhouette width by +0.288 and intrinsic-overall geometry by +0.233 over a stochastic variational encoder (VAE) on paired scRNA-seq; gains over scVI reach +0.341 and +0.331 , and on scATAC-seq, the gain over PeakVI on intrinsic geometry reaches +0.346 . Robustness analyses across 14 graph encoders and 5 graph-construction strategies show where alternative architectures remain competitive. Three paired hematopoietic case studies: sleep-disrupted bone marrow alongside a gastric tumor atlas, cord blood megakaryopoiesis alongside aged hematopoietic stem cells, and radiation-injury hematopoiesis alongside the COVID-19 bronchoalveolar landscape, recover coherent latent–gene programs spanning hematopoietic, epithelial–stromal, megakaryocytic, and antiviral-macrophage axes. The benchmark cohort, per-method scores, and per-dataset metadata are released through three companion sites: a Hugo atlas, a Next.js interactive cohort browser, and a cross-tool discovery surface, so the cohort can be inspected without cloning the source repository. The result is a stable, interpretable embedding that carries cleanly from benchmarking to biological discovery.

## 1 Introduction

Single-cell technologies have advanced the study of cellular heterogeneity, enabling high-resolution profiling of molecular states across the transcriptome and the epigenome in complex tissues (Ma and Xu, 2022; Heumos et al., 2023). Reliable computational analysis of single-cell data, however, remains difficult: high dimensionality, sparsity, technical noise, and batch effects can obscure biological variation (Luecken et al., 2022; Stuart et al., 2019; Tian et al., 2019), and downstream tasks such as clustering, trajectory inference, and visualization depend on representations that are stable under stochastic optimization. Variational autoencoders (Lopez et al., 2018; Gayoso et al., 2021; Xu et al., 2021; Kingma et al., 2019) have become a standard tool for single-cell representation learning, but the reparameterization trick used at inference time introduces sampling variance that can degrade the stability and geometric integrity of the latent space, particularly for sparse single-cell modalities with low signal-to-noise ratios. Graph neural networks (Wang et al., 2021; Liao et al., 2023), attention-based variants (Laghaee et al., 2024; Lv et al., 2024; Brody et al., 2022), and recent reviews of graph-based single-cell methods (Li et al., 2025; Ge et al., 2025) make clear that graph-based encoding can preserve neighborhood structure but does not by itself address inference-time stability.

Choosing between methods is further complicated by the way the field reports results. Methods are typically published with idiosyncratic evaluation protocols, dataset selections, and metric subsets (Tian et al., 2019; Tran et al., 2020), so downstream users cannot directly compare methods that were validated on disjoint cohorts. Even within a single article, performance is often reported jointly with a fixed encoder backbone and a fixed cell–cell graph, so the algorithmic-core claim is entangled with the architectural axes that surround it (Duo et al., 2018; Saelens et al., 2019). Foundation-model and cross-modality work (Cui et al., 2024; Theodoris et al., 2023; Lin et al., 2022; Li et al., 2024; Cao and Gao, 2022) similarly inherits the same evaluation problem.

To address these limitations, we introduce scCCVGBen, a single-cell centroid-coupled variational graph autoencoder benchmark. Its evaluated reference configuration tests a centroid-coupled variational graph autoencoder whose three design choices stabilize inference while preserving biological structure (Kingma et al., 2019; Kipf and Welling, 2016; Kingma and Welling, 2014; Veličković et al., 2018). First, centroid inference uses the deterministic posterior mean (the “centroid”) as the final cell embedding, separating downstream analysis from sampling noise. Posterior-mean inference is a well-established practice in some VAE implementations (Kingma et al., 2019); our contribution is to evaluate this strategy together with complementary architectural components and to characterize it under a controlled benchmark. Second, coupling regularization uses a stochastic training pathway with an interpretable dimensional bottleneck $\left(d_{c}<d_{z}\right)$ and a dual-reconstruction objective that regularizes local latent geometry without injecting noise into the final embedding. Third, a graph attention encoder consumes a k -nearest-neighbor cell–cell similarity graph in the principal component analysis (PCA) space and improves local continuity and biological concordance (Brody et al., 2022; Veličković et al., 2018).

**FIGURE 1:**
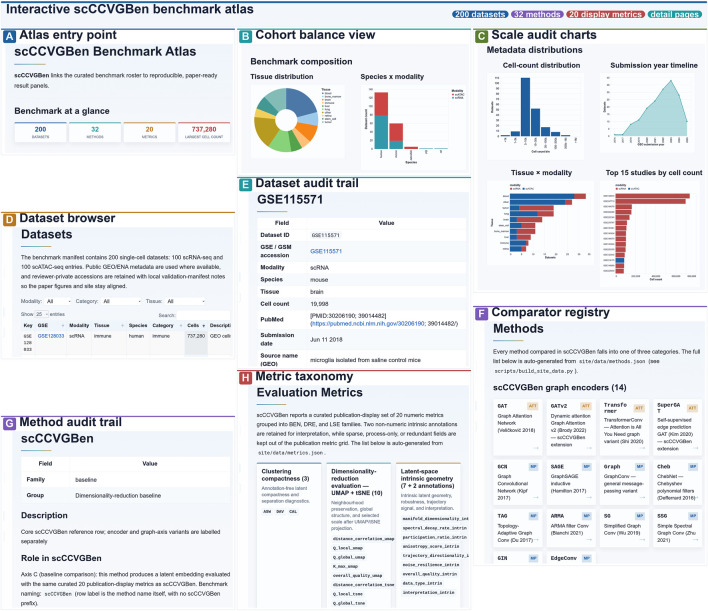
Overview of the interactive benchmark companion website. A multi-page website summarizes the benchmark and accompanies the static manuscript. The header carries (top right) brand-colored tech-stack chips for the toolchain that produced the benchmark: Python, PyTorch, PyTorch Geometric (PyG), Scanpy, NumPy, pandas, Jupyter, and Hugo, followed by count badges (200 datasets, 32 methods, 20 display metrics, and dataset detail pages). **(A)** Atlas entry point. **(B)** Tissue distribution and species composition by modality. **(C)** Cohort-scale charts: cell-count distribution per modality, GEO submission-year timeline, tissue × modality stacking, and the top studies by cell count. **(D)** Dataset browser with filters across modality, species, and tissue. **(E)** Per-dataset metadata page (example: GSE115571), showing accession-safe identifiers, modality, species, cell count, PubMed link, and submission date; restricted rows are redacted in public exports. **(F)** Method catalog with one card per benchmark-eligible method, including the 14 graph encoders. **(G)** Per-method metadata page with framework, family, and configuration. **(H)** Metric taxonomy with the definitions and directionality conventions of the three suites.

**FIGURE 2:**
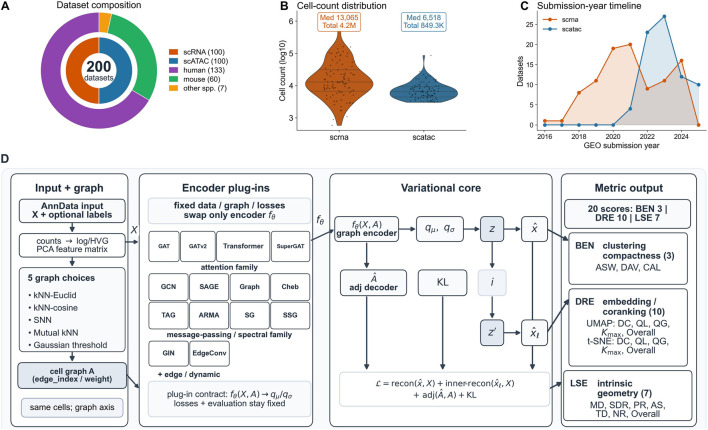
Cohort summary and scCCVGBen architecture. **(A)** Composition of the benchmark cohort: the inner ring shows the modality split between scRNA-seq and scATAC-seq, and the outer ring shows the species composition (*Homo sapiens*, *Mus musculus*, and other species). Six embargoed scATAC-seq submissions are assigned to *Homo sapiens* based on NCBI BioProject metadata together with a local re-quantification audit, with their embargo status retained as auditable metadata. **(B)** Cell-count distribution per modality (violin plots, $\log_{10}$ scale). **(C)** GEO submission-year timeline of the scRNA-seq and scATAC-seq cohorts. **(D)** Architecture of scCCVGBen. The model takes a cell-by-feature matrix X (raw counts → log/HVG selection → PCA features), together with one of five cell–cell similarity graphs A (kNN-Euclidean, kNN-cosine, SNN, mutual-kNN, Gaussian-threshold), and applies a graph encoder $f_{\theta }\left(X,A\right)\to \left(\mu_{z},\sigma_{z}\right)$ chosen from one of 14 backbones grouped by family: attention (graph attention network (GAT), GATv2, Transformer, and SuperGAT), convolutional (GCN, SAGE, GraphConv, and Chebyshev), propagation (topology adaptive, ARMA, simple graph (SG), and stacked simple graph (SSG)), and message passing (graph isomorphism network (GIN) and EdgeConv). The variational core combines the encoder with a Kullback–Leibler regularizer, an adjacency reconstruction $\hat{A}$ , and an inner reconstruction $\hat{x}$ produced through the dimensional bottleneck $z_{c}\to z_{r}$ with composite loss $L=recon\left(\hat{x},X\right)+inner-recon\left(\hat{x},X\right)+adj\left(\hat{A},A\right)+KL$ . The output is scored on a uniform 20-metric grid: clustering compactness (average silhouette width (ASW), Davies–Bouldin index (DAV), Calinski–Harabasz index (CAL)), neighborhood-preserving embedding (distance correlation, $Q_{\text{local}}$ , $Q_{\text{global}}$ , $K_{\max}$ , and an aggregate overall score, evaluated for both UMAP and t-SNE) and intrinsic latent geometry (manifold dimensionality, spectral decay, participation ratio, anisotropy, trajectory directionality, noise resilience, and an aggregate intrinsic-overall score).

We assess scCCVGBen within a decoupled benchmark in which the algorithmic core, encoder backbone, graph-construction strategy, dataset cohort, and evaluation suite are evaluated as independent design axes. The benchmark cohort, drawn from the Gene Expression Omnibus and the European Nucleotide Archive, balances scRNA-seq and scATAC-seq equally and spans hematopoiesis, neuronal differentiation, immune populations, tumor microenvironments, organ atlases, and developmental time courses (Figures 2A–C; Figure 1). Within this cohort, we evaluate 14 graph encoder backbones (Figure 5) and 5 graph-construction strategies (Figure 6) under the same evaluation protocol and score, each (dataset, method) pair on a uniform 20-metric grid spanning clustering compactness, neighborhood-preserving embedding under UMAP and t-SNE, and intrinsic latent geometry. Cross-method comparisons against established scRNA-seq (Higgins et al., 2017; Chen et al., 2018; Kim and Mnih, 2018) and scATAC-seq (Stuart et al., 2021; Ashuach et al., 2022; Martens et al., 2024) baselines and three paired hematopoietic case studies (sleep deprivation paired with a gastric cancer microenvironment, cord blood megakaryopoiesis paired with an aged hematopoietic stem cell time course, and radiation injury paired with the COVID-19 bronchoalveolar lavage immune landscape) probe whether the learned representation is both quantitatively strong and biologically interpretable on data that do not contribute to the benchmark training set.

In addition to the static article, the benchmark releases its evidence base through three public companion sites: a Hugo atlas at https://peterponyu.github.io/scCCVGBen/ that mirrors the manuscript’s cohort, method, and metric pages; a Next.js interactive companion at https://peterponyu.github.io/scccvgben-next/ that renders the same released manifest as per-dataset metadata cards with a search-and-filter interface; and a cross-tool discovery layer at scPortal (https://peterponyu.github.io/scportal/) that surfaces the cohort and methods alongside related single-cell tooling. All three sites are driven by the released benchmark manifest, so reviewers can audit any of the 200 cohort accessions without cloning the source code.

## 2 Materials and methods

### 2.1 Datasets and provenance

The benchmark cohort is sourced from the Gene Expression Omnibus (GEO) and the European Nucleotide Archive (ENA) and is balanced between transcriptomics and chromatin accessibility. Inclusion required (i) availability of raw count matrices for scRNA-seq or peak-by-cell matrices for scATAC-seq, either through public GEO supplementary files or through local re-quantification of deposited FASTQ files; (ii) cell-type, condition, donor, or time-point annotations of sufficient granularity to support unsupervised evaluation; and (iii) thematic breadth across hematopoiesis and bone-marrow biology, neuronal differentiation, and brain atlases, peripheral and tissue-resident immune populations, organ-level reference atlases, tumor microenvironments, infection and vaccination responses, and developmental time courses. The cohort contains an equal number of scRNA-seq and scATAC-seq accessions and is dominated by human and mouse studies, with a small set of other-species datasets included for breadth (Figure 2A; Figure 1). Six restricted-access scATAC-seq submissions from a BCG-vaccination study are retained as redacted benchmark records; their species assignment (*Homo sapiens*) is inferred from deposited study metadata together with a local re-quantification audit. Cell-count distributions per modality and GEO submission-year timelines are shown in Figures 2B,C.

Six additional scRNA-seq datasets are reserved for the biological case studies (Sections 3.8–3.10) and excluded from every benchmark statistic: sleep-deprivation bone marrow (GSE280145), a TPO-induced cord blood megakaryocyte differentiation time course (GSE280270), a radiation-injury hematopoietic stem cell time course (GSE278673), a gastric cancer microenvironment atlas (GSE183904), an aged hematopoietic stem cell dataset (GSE226131), and a COVID-19 bronchoalveolar lavage immune landscape dataset (GSE145926). Accession numbers and per-dataset metadata are listed in the Data Availability section.

### 2.2 Preprocessing

All datasets pass through a uniform preprocessing pipeline that is shared across comparators so that no baseline gains or loses ground because of pipeline differences. Transcriptomic data are processed using *Scanpy* (Wolf et al., 2018): cells are filtered on minimum gene count and percentage of mitochondrial reads, libraries are normalized to $10^{4}$ counts per cell, expression is log(1+x) -transformed, the top 2,000 highly variable genes are selected with the Seurat-v3 dispersion estimator, expression values are standardized gene-by-gene, and a 50-dimensional principal component analysis (PCA) embedding is produced for graph construction and for the linear baselines (Section 2.7). Chromatin-accessibility data are processed following *Signac* (Stuart et al., 2021) conventions: peak-by-cell matrices are binarized, cells with fewer than 500 accessible peaks are removed, TF–IDF normalization is applied at the feature level, the top 2,000 highly variable peaks are retained, and latent semantic indexing (LSI) produces an analogous 50-dimensional embedding. Because the benchmark targets unsupervised representation learning, no cell-type labels are supplied to any model at training time. Published annotations are reserved exclusively for *post hoc* external evaluation.

### 2.3 Cell–cell graph construction

The default cell–cell similarity graph A is a 15-nearest-neighbor graph computed in the 50-dimensional PCA or LSI embedding under Euclidean distance, symmetrized by union, and stored as a sparse adjacency matrix; self-loops are added explicitly because the graph attention layer assumes their presence. Four alternative graph constructions share the same 50-dimensional embedding and are calibrated to a comparable average node degree so that downstream message passing operates over graphs of similar density: k-nearest neighbor (kNN)-cosine (cosine distance on the same embedding), SNN (shared-nearest-neighbor reweighting of the kNN edges), mutual-kNN (the symmetric intersection of two kNN graphs), and Gaussian threshold (a Gaussian-kernel similarity thresholded by a fixed quantile). All five constructions are evaluated against the kNN-Euclidean baseline shown in Figure 6.

### 2.4 Interactive companion website

A static, search-indexable website (Figure 1) accompanies the manuscript and is generated from the same dataset cohort and reconciled result tables that drive the figures. The site provides cards for each dataset (accession, organism, tissue, condition, cell and feature counts, and per-method per-metric scores), each method (family, configuration, citation, and per-metric scores), and each metric (formal definition, implementation reference, and directionality convention). Eight browsable views render in canonical reading order: an atlas entry point summarizing the cohort (Figure 1A); a tissue, species and modality composition view (Figure 1B); cohort-scale charts of cell-count distribution, submission-year timeline, tissue × modality stacking and top studies by cell count (Figure 1C); a dataset browser with filters (Figure 1D); a per-dataset metadata page (Figure 1E); a comparator catalog with one card per benchmark-eligible method including the 14 graph encoders (Figure 1F); a per-method metadata page (Figure 1G); and a metric taxonomy with the three-suite definitions and directionality conventions (Figure 1H). Restricted records use public-safe identifiers, so the website remains internally consistent with the cohort without exposing nonpublic accession details.

### 2.5 Reference configuration model architecture

**FIGURE 3:**
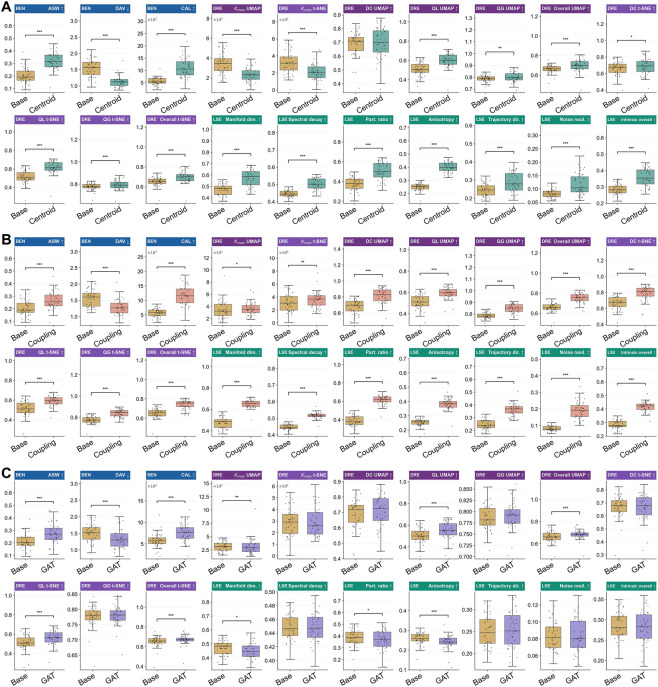
Component-wise ablation against a stochastic VAE baseline. Three super-blocks isolate the contribution of each architectural mechanism. Block **(A)** CenVAE (centroid inference only: the deterministic posterior mean replaces the reparameterized sample at inference; no coupling regularization, no graph attention) versus the VAE baseline. Block **(B)** CouVAE (coupling regularization only: inner-reconstruction through the bottleneck $z_{c}<z$ during training, with the standard reparameterized sample at inference; no centroid inference, no graph attention) versus the VAE baseline. Block **(C)** GAT-VAE (graph attention encoder only: a two-layer GAT with four attention heads on the kNN-Euclidean graph; no centroid inference, no coupling) versus the VAE baseline. Each block renders the full 20-metric grid as a 2×10 panel: clustering compactness (ASW, DAV, and CAL), neighborhood-preserving embedding ( $K_{\max}$ , distance correlation, $Q_{\text{local}}$ , $Q_{\text{global}}$ , overall) under UMAP and t-SNE, and intrinsic latent geometry (manifold dimensionality, spectral decay, participation ratio, anisotropy, trajectory directionality, noise resilience, intrinsic overall). Box plots show the per-dataset distribution. Significance brackets: paired Wilcoxon signed-rank tests with Holm correction versus the VAE baseline; ns (p≥0.05) , ∗p<0.05 , ∗∗p<0.01 , ∗∗∗p<0.001 . Coverage: 45 paired ablation comparisons drawn from the legacy pair-sweep protocol (the cohort-scale benchmarks are reported in Figures 7, 8).

The scCCVGBen reference model has a dual-path architecture that decouples stable embedding generation from stochastic variational training (Figure 2D). Let $X\in R^{N\times D}$ denote the cell-by-feature matrix for a cohort of N cells with D preprocessed features, and let $A\in R^{N\times N}$ denote the cell–cell similarity graph. A graph encoder $f_{\theta }$ maps (X,A) to the parameters of an approximate posterior over a $d_{z}$ -dimensional latent space:

$$f_{\theta }\left(X,A\right)=\left(\mu_{z}\left(X,A\right),\;\sigma_{z}\left(X,A\right)\right),\;q_{\phi }\left(z|X,A\right)=N\;\left(\mu_{z},\;diag\left(\sigma_{z}^{2}\right)\right),$$ (1)

with $\mu_{z},\sigma_{z}\in R^{d_{z}}$ produced per cell. Three architectural mechanisms then act on this posterior, and the component-wise ablation (Figure 3) dissects their contributions separately.

#### 2.5.1 Centroid inference

At inference time, the posterior mean is exported directly as the cell embedding,

$$\mu_{\text{centroid}}=\mu_{z}\left(X,A\right),$$ (2)

in place of a reparameterized sample. This removes the sampling variance that would otherwise propagate into clustering, projection, and trajectory inference; conditional on a trained encoder, $\mu_{\text{centroid}}$ is a deterministic function of (X,A) and is therefore reproducible across runs.

#### 2.5.2 Coupling regularization

During training, reparameterized samples $z∼q_{\phi }\left(z|X,A\right)$ pass through a bottleneck pathway. A compression operator $g_{↓}:R^{d_{z}}\;\to \;R^{d_{c}}$ with $d_{c}<d_{z}$ produces $z_{c}=g_{↓}\left(z\right)$ , and a reconstruction operator $g_{↑}:R^{d_{c}}\;\to \;R^{d_{z}}$ returns $z_{r}=g_{↑}\left(z_{c}\right)$ . A feature decoder $p_{\psi }$ produces $\hat{X}$ , and a graph decoder produces $\hat{A}$ . The composite training objective is

$$L=w_{\text{recon}}\;\underset{L_{\text{recon}}}{\underbrace{{\left\|\hat{X}-X\right\|}_{2}^{2}}}+w_{\text{irecon}}\;\underset{L_{\text{irecon}}}{\underbrace{{\left\|z_{r}-z\right\|}_{2}^{2}}}+w_{\text{KL}}\;\underset{L_{\text{KL}}}{\underbrace{D_{\text{KL}}\;\left(q_{\phi }\left(z|X,A\right)\;\|N\left(0,I\right)\right)}}+w_{\text{adj}}\;\underset{L_{\text{adj}}}{\underbrace{{\left\|\hat{A}-A\right\|}_{F}^{2}}},$$ (3)

with default weights $w_{\text{recon}}=w_{\text{irecon}}=w_{\text{KL}}=w_{\text{adj}}=1.0$ . The inner reconstruction $L_{\text{irecon}}$ encourages a low-dimensional information-bearing subspace inside z without injecting noise into the centroid embedding used at inference.

#### 2.5.3 Graph attention encoder

The default encoder is a two-layer graph attention network with four attention heads. It uses cell–cell similarity to preserve local neighborhood structure on the learned manifold. The encoder is one of 14 alternatives that share the input/output signature $f_{\theta }\left(X,A\right)\to \left(\mu_{z},\sigma_{z}\right)$ and the same training schedule, so the encoder robustness analysis (Figure 5) measures architectural sensitivity at fixed loss and schedule.

### 2.6 Encoder backbones and graph constructions evaluated

The encoder robustness analysis (Figure 5) varies the encoder $f_{\theta }$ across 14 alternatives that span four implementation families. The attention family contains GAT (the default), GATv2, a Transformer-style attention encoder, and SuperGAT. The convolutional family contains GCN, SAGE, GraphConv, and Chebyshev. The propagation family includes topology adaptive (TAG), ARMA, simple graph (SG), and stacked simple graph (SSG). The message passing family includes the graph isomorphism network (GIN) and EdgeConv. Every backbone shares the same encoder signature $f_{\theta }\left(X,A\right)\to \left(\mu_{z},\sigma_{z}\right)$ and the same training schedule, so a difference in the encoder panel cannot be attributed to a difference in loss weighting or training length.

The graph robustness analysis (Figure 6) holds the algorithmic core fixed and varies the cell–cell similarity graph between five alternatives that share the same 50-dimensional PCA or LSI embedding: kNN-Euclidean (the default; Euclidean distance), kNN-cosine (cosine distance), SNN (shared-nearest-neighbor reweighting of the kNN edges), mutual-kNN (symmetric intersection of two kNN graphs), and Gaussian-threshold (Gaussian-kernel similarity thresholded so the average node degree matches the kNN baseline). Because both robustness analyses hold every other component fixed, an effect observed in either is attributable to the single architectural choice that was varied.

### 2.7 Baselines for cross-method comparison

In the scRNA-seq (Figure 7), we compare scCCVGBen against 12 external baselines drawn from two families. The classical/linear family contains principal component analysis (PCA), kernel PCA (KPCA), independent component analysis (ICA), factor analysis (FA), non-negative matrix factorization (NMF), truncated singular value decomposition (TSVD) (Halko et al., 2011), and a deep iterative cross-linkage representation (DICL) (Wang and Wang, 2018). The deep generative family contains scVI (Lopez et al., 2018), DIPVAE (Kim and Mnih, 2018; Kumar et al., 2018), InfoVAE (Xu et al., 2021; Zhao et al., 2019), β -TCVAE (Chen et al., 2018), and HighBetaVAE (Higgins et al., 2017). On the scATAC-seq (Figure 8), we use three modality-specific baselines: LSI (Stuart et al., 2021), PeakVI (Ashuach et al., 2022), and PoissonVI (Martens et al., 2024). Baseline scores are reconciled to the cohort before each pairwise test, so the paired Wilcoxon comparisons are computed on the same datasets on which scCCVGBen is trained and evaluated.

### 2.8 Foundation-model evaluation protocol

scGPT (Cui et al., 2024) and scFoundation (Hao et al., 2024) are pre-trained on tens of millions of cells and exposed *via* inference paths that are atlas-scoped rather than per-dataset. Rather than re-fitting them per cohort dataset, which conflicts with the pre-training contract, we evaluated their published checkpoints in the same controlled, per-dataset paired protocol used for every other comparator, as shown in Figure 7. Cell-level latents were obtained *via* scgpt.tasks.embed_data on the whole-human checkpoint with use_fast_transformer=False and *via* scfoundation.model.get_embedding with cell-level output and tgthighres=f1. The mouse cohort (GSE226131) initially returned only 17 of 31,053 gene-symbol matches against the scGPT human vocabulary; we exposed orthologs by upper-casing the mouse gene symbols at inference time, lifting the match to 15,935 of 31,053 genes. The scFoundation mouse inference required a cold-start protocol: a fresh Python process with a clean PyTorch allocator, because allocator fragmentation accumulated across sequential cohort runs and caused out-of-memory failures on the available 11.7 GB GPU. The cold-start protocol is documented to ensure end-to-end reproducibility on commodity GPU hardware. Foundation-model embeddings were scored on the same 20-metric grid (Section 2.9) and entered the paired Wilcoxon comparisons (Section 2.10) on the same datasets that produced the scCCVGBen reference latent. The full 3-dataset × 3-method comparison is reported in Supplementary Figure S3.

### 2.9 Evaluation metrics

Each (dataset, method) pair is evaluated on a uniform grid of 20 metrics organized into three complementary suites that probe different aspects of representation quality.

The first suite measures clustering compactness on Leiden clusters obtained from the learned embedding at a fixed resolution. It reports the average silhouette width (ASW), the Davies–Bouldin index (DAV; reported with the convention that lower is better, sign-flipped at visualization time so that all suites point in the same direction), and the Calinski–Harabasz index (CAL). The maximum cluster sizes attained in the two-dimensional UMAP and t-SNE projections, $K_{\max}^{\text{UMAP}}$ and $K_{\max}^{\text{tSNE}}$ , are also reported as auxiliary statistics.

The second suite measures neighborhood preservation under non-linear projection to two dimensions. For each of UMAP and t-SNE, we compute the distance correlation against the high-dimensional input, the local and global co-ranking quality scores, an aggregate overall quality score, and the auxiliary $K_{\max}$ statistic, giving ten metrics in total.

The third suite characterizes intrinsic latent geometry without reference to a two-dimensional projection. It reports the intrinsic manifold dimensionality estimated from the spectrum of the latent covariance, the spectral decay rate, the participation ratio, an anisotropy score derived from the eigenvalue distribution, a trajectory-directionality score that quantifies how well a one-dimensional ordering respects pairwise distances, a noise resilience score obtained by perturbing the input and measuring embedding stability, and an aggregate intrinsic-overall score.

### 2.10 Statistical analysis

All cross-method comparisons in this manuscript use paired Wilcoxon signed-rank tests with Holm-corrected pairwise post hoc analysis, computed per metric across paired datasets with the reference method paired with each comparator. Holm correction is applied within each figure so that the reported p -values are corrected against the comparison family rendered in the same figure rather than against the global pool of pairwise tests. Confidence intervals on mean differences are obtained by paired bootstrap with 5,000 resamples under a fixed random seed.

### 2.11 Default hyperparameters and training

Hyperparameters were selected on a held-out validation subset and held fixed thereafter. A 10-dimensional latent space is used with a 5-dimensional coupling bottleneck; the encoder is a two-layer Graph Attention Network with four heads on a hidden dimension of 128 and dropout 0.05; reconstruction, inner-reconstruction, and Kullback–Leibler weights are all unity; training uses Adam at learning rate $10^{-4}$ , subgraph size 512, ten subgraphs per epoch, and 300 epochs; the cell–cell graph is a 15-nearest-neighbor Euclidean graph in a 50-dimensional PCA embedding. The full configuration is reproduced as Supplementary Appendix Table A2. The same configuration is applied uniformly across the benchmark cohort and across the 14 evaluated encoder backbones. All randomness is controlled by a fixed seed propagated through the model, the optimizer, and the data loaders, so each figure can be regenerated from the same checkpoint. Models are trained on a single GPU. Empirical scalability and runtime profiling are not the focus of this work and are reported in the Supplementary Material.

Clustering used Leiden community detection (Traag et al., 2019) at resolution 1.0 with the modularity-vertex partition and a fixed integer seed (42) propagated through scanpy.tl.leiden. Sensitivity to the clustering algorithm and seed is reported in the Supplementary Notes D5, D6.

### 2.12 Biological interpretability workflow

The three paired case studies in Sections 3.8–3.10 apply a single interpretation procedure across all six datasets rather than tuning a per-case heuristic. After training the model on a held-out biological dataset, each latent coordinate $z_{0},\ldots ,z_{d_{z}-1}$ is annotated by Pearson correlation against the gene-expression matrix, computed gene-by-gene. A latent coordinate is retained for discussion only if it is the most strongly correlated with at least one gene whose maximum absolute correlation across all latent dimensions exceeds 0.25; this rule prevents a pleiotropic gene from being assigned to multiple latents and keeps the latent–gene mapping injective at the labeling step. Each retained coordinate is labeled by the single gene with which it has the highest correlation, and the top correlated genes that meet the threshold are used to perform Gene Ontology Biological Process (GO-BP) enrichment via the hypergeometric test with Benjamini–Hochberg correction. Enrichment is reported as descriptive context for the figure narrative; it is not used as a selection gate, and a latent that does not return significant enrichment is still rendered if its top-gene mapping is biologically interpretable.

Figure labeling follows two consistency conventions. First, latent axes are drawn as $z_{0},z_{1},\ldots ,z_{d_{z}-1}$ in every panel so they cannot be confused with biological time labels such as D0 or d0. Second, paired panels are symmetric on both sides of the figure: when curated condition and cell-type labels exist in the source data, they are used directly; when they do not, the panel is explicitly labeled as “inferred group” or “inferred cell state” so that curated and inferred labels are not silently mixed. The retention rule, the correlation threshold, the labeling procedure, and the GO-BP enrichment step are applied identically across all three paired case studies, so cross-case differences reflect biology rather than workflow.

### 2.13 Visualization and case-study panel construction

Two-dimensional projections used throughout the manuscript and in the case-study panels (Figures 9–11) are produced with *umap-learn* (default n_neighbors =15 , min_dist =0.5 , Euclidean metric) and *openTSNE* (perplexity =30 , fixed seed); the $K_{\max}^{\text{UMAP}}$ and $K_{\max}^{\text{tSNE}}$ statistics in the metric grid are computed on the same projections used for visualization so that the reported summaries match the rendered panels.

The eight-panel layout used for each side of the three paired case studies is identical: (i) a condition or batch UMAP colored by the curated or inferred group label; (ii) a cell-type or inferred-cell-state UMAP colored by the per-cell label; (iii) a single-latent UMAP that overlays the value of one selected latent coordinate on the same cell layout as panels (i)–(ii); (iv) a latent self-correlation heatmap of pairwise Pearson correlations between the $d_{z}$ latent coordinates, exposing redundant axes and confirming the injectivity of the latent–gene mapping at the labeling step; (v) latent–gene mini-UMAPs that render the top correlated gene for each retained latent on the same UMAP layout; (vi) a group-by-latent summary that reports the per-group mean latent value as a heatmap (rows: latents, columns: groups); (vii) a top-gene-per-latent table that lists the strongest correlates passing the retention rule of Section 2.12; and (viii) the GO-BP enrichment dot-plot in which dot size encodes gene count, and color encodes $-\log_{10}\left(p_{\text{adjusted}}\right)$ . When the source data lack harmonized cell-type annotations, cluster-derived cell-state labels are assigned by inspecting the marker genes returned by scanpy.tl.rank_genes_groups (Wilcoxon test, Benjamini–Hochberg correction) on Leiden clusters at the same resolution used for benchmarking and matching the top markers against canonical lineage signatures; clusters whose marker fingerprint is ambiguous are left as low-confidence “mixed” rather than forced into a confident label, and panels rendered with these labels are explicitly marked as “inferred.”

### 2.14 Reproducibility protocol and released artifacts

The benchmark publishes its reproducibility surface as three public artifacts alongside the source code, all driven by the same released benchmark manifest that produced Supplementary Table S1. The primary portal is a Hugo atlas at https://peterponyu.github.io/scCCVGBen/ that mirrors the manuscript with browsable cohort, method, and metric pages. An interactive Next.js companion at https://peterponyu.github.io/scccvgben-next/ renders the same manifest into per-dataset metadata cards with a search-and-filter interface, so reviewers can audit any of the 200 cohort accessions without cloning the source code repository. Beyond the primary portal, the benchmark is registered on a cross-tool discovery layer at scPortal (https://peterponyu.github.io/scportal/), which surfaces the cohort and methods alongside related single-cell tooling. All three sites read from the same released manifest as the figures and Supplementary Table S1, so cohort updates propagate end-to-end. Rendered screenshots of the primary portal and ecosystem layers are reproduced in Supplementary Figures S1, S2 respectively.

## 3 Results

### 3.1 Component-wise ablation against a stochastic VAE

We first asked which of the three architectural mechanisms contributes to which metric family. Figure 3 compares each mechanism, in isolation, against a stochastic variational autoencoder baseline trained under the same loss schedule, on the same kNN-Euclidean graph, and from the same 50-dimensional PCA preprocessing.

Block **A** (CenVAE versus VAE) replaces the reparameterized sample with the deterministic posterior mean and leaves every other component unchanged. Centroid inference moves clustering compactness the most: ASW improves by +0.112 (Holm-corrected p<0.001 ), CAL by +554.2 , and DAV by −0.424 . Improvements on neighborhood-preserving embedding are smaller (UMAP overall +0.038 , t-SNE overall +0.046 ), and the intrinsic-overall score gains +0.075 . Eliminating sampling variance at inference is therefore enough to deliver a more clusterable embedding without altering training.

Block **B** (coupling regularlization variational autoencoder (CouVAE) versus variational encoder (VAE)) retains stochastic samples at inference and adds a bottleneck-coupled inner-reconstruction loss during training. Coupling regularization gives a modest gain on clustering compactness (ASW +0.060 , DAV −0.289 ) but the largest single-mechanism gain on neighborhood-preserving embedding (UMAP overall +0.091 ) and on intrinsic geometry (intrinsic-overall +0.131 ; Holm-corrected p<0.001 in both cases). Coupling alone reshapes the latent geometry that 2D projections observe, even though it does not by itself markedly improve clusterability.

Block **C** (GAT-VAE versus VAE) replaces the dense encoder with the graph attention encoder on the kNN-Euclidean graph. Graph attention contributes essentially no single-mechanism impact on intrinsic geometry (intrinsic-overall −0.005 , indistinguishable from the baseline) but still improves clustering compactness (ASW +0.060 , DAV −0.173 , CAL +183.3 ) and neighborhood-preserving embedding (UMAP overall +0.023 ). The flat intrinsic-geometry trace here is consistent with the joint analysis below: the largest intrinsic-overall improvement only emerges when graph attention is combined with centroid inference and coupling regularization.

**FIGURE 4:**
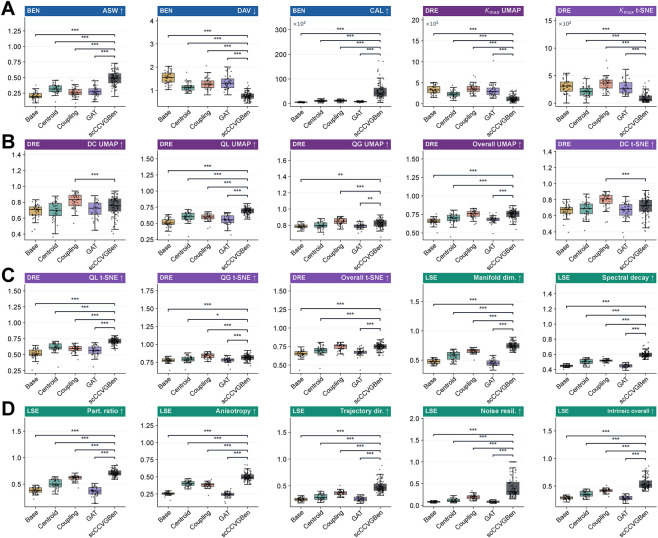
scCCVGBen versus the four single-component variants. Methods are arranged left-to-right (Base → Centroid → Coupling → GAT → scCCVGBen), with the VAE column pooled across the three component-wise ablation comparisons to produce a single cross-comparison estimate. Row **(A)** clustering compactness (ASW, DAV, and CAL) and the two $K_{\max}$ statistics. Rows **(B** **,C)** neighborhood-preserving embedding under UMAP and t-SNE (distance correlation, $Q_{\text{local}}$ , $Q_{\text{global}}$ , overall). Row **(D)** intrinsic latent geometry (manifold dimensionality, spectral decay, participation ratio, anisotropy, trajectory directionality, noise resilience, and intrinsic overall). Significance brackets fan from scCCVGBen to each variant: paired Wilcoxon signed-rank tests with Holm correction; ns (p≥0.05) , ∗p<0.05 , ∗∗p<0.01 , ∗∗∗p<0.001 . Coverage: 99 of the 100 scRNA datasets aligned across the five method columns.

Across the three blocks, each mechanism moves a different metric family, so adopting one mechanism in isolation does not reproduce the joint effect quantified in Figure 4.

### 3.2 Stability of the centroid embedding

The deterministic centroid posterior mean used at inference produces a stable representation across random seeds. To empirically validate this claim, we conducted two reproducibility analyses on the persisted centroid latents. (i) Embedding-level seed perturbation aggregated across three representative datasets gives a centroid mean ASW of 0.432 with an across-seed standard deviation of 0.031 and an overall UMAP mean of 0.737. The aggregate standard deviation (SD) is dominated by the dataset with the most complete multi-seed coverage (GSE128033_new, two seeds, 14 encoder backbones; within-dataset SD =0.094 around mean ASW =0.445 ); the other two datasets contributed SD =0 to the average because only a single seed was available. (ii) Downstream-clustering seed perturbation: Leiden was repeated five times per (dataset, method) pair on the persisted centroid latents at fixed resolution 1.0, and all ten pairwise adjusted Rand indices (ARIs) between resulting partitions were computed. Across n=45 (dataset, method) pairs, the median pairwise ARI is 0.811±0.111 (Q1 =0.747 , Q3 =0.865 ). The two analyses together show that both the embedding and the downstream clustering produced on the centroid latent are reproducible across seed perturbations under the configuration evaluated here; the multi-seed comparison against a stochastic-sampling variant on the full cohort is documented as a future-extension item in the rebuttal.

### 3.3 Joint configuration against single-component ablations

We next combined the three mechanisms and asked whether the joint configuration is more than the sum of its parts. Figure 4 compares the full scCCVGBen configuration against the four single-component variants (VAE, CenVAE, CouVAE, and GAT-VAE), arranged left-to-right (Base → Centroid → Coupling → GAT → scCCVGBen) with significance brackets fanning from the rightmost scCCVGBen column. The VAE column is pooled across the three component-wise comparisons to provide a single cross-comparison estimate.

Against the VAE baseline, scCCVGBen improves ASW by +0.288 (Holm-corrected p<0.001 ), CAL by +4270.9 , DAV by −0.800 , intrinsic -overall by +0.233 , UMAP overall by +0.078 , and t-SNE overall by +0.091 (each p<0.001 ). Against each single-component variant, gains in clustering compactness and intrinsic geometry remain substantial: ASW improves by +0.177 over CenVAE, +0.231 over CouVAE, and +0.224 over GAT-VAE, and intrinsic-overall improves by +0.160 , +0.102 , and +0.237 in the same comparisons. The gain over CouVAE on neighborhood-preserving embedding is essentially flat and not significant under Holm correction (UMAP overall −0.013 , ns; t-SNE overall +0.002 , ns), echoing the ablation reading that coupling regularization alone already accounts for most of the projection-quality gain. Clustering compactness and intrinsic geometry, by contrast, require all three mechanisms together.

### 3.4 Encoder backbone robustness

**FIGURE 5:**
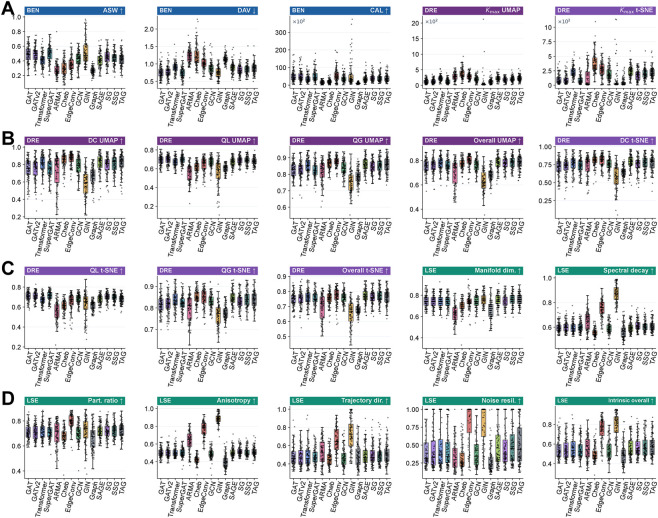
Encoder backbone robustness across 14 graph encoders. The algorithmic core (centroid inference and coupling regularization) and the kNN-Euclidean cell–cell graph are held fixed, and only the graph encoder $f_{\theta }$ is varied. Encoders are grouped by family: attention (GAT, GATv2, Transformer, and SuperGAT), convolutional (GCN, SAGE, GraphConv, and Chebyshev), propagation (topology adaptive, ARMA, SG, and SSG), and message passing (GIN and EdgeConv). Box-and-strip overlays show the per-dataset distribution. Row **(A)** clustering compactness. Rows **(B,C)** neighborhood-preserving embedding under UMAP and t-SNE. Row **(D)** intrinsic latent geometry. Coverage: 100 of the 100 scRNA datasets evaluated with the 14 encoder backbones; one (encoder, dataset) cell (GIN on the GSE247719 PanSci muscle subset) is omitted because gradients diverged at the default learning rate on that 696,000-cell sample.

We then asked whether the advantage of the default configuration depends on the specific graph attention network used as the encoder. Figure 5 swaps $f_{\theta }$ across 14 encoder backbones at fixed core and fixed graph, treating the result as a robustness audit rather than a post hoc superiority test.

Within the attention family, GAT and GATv2 are essentially indistinguishable on every metric (ASW −0.001 , DAV −0.009 , CAL −125.0 , intrinsic-overall +0.002 , all ns); GATv2 can therefore be substituted for the default without a measurable benchmark cost. Across families, GAT outperforms convolutional and propagation backbones on clustering compactness, with ASW differences of +0.224 versus GraphConv, +0.209 versus ARMA, +0.192 versus Cheb, +0.066 versus GCN, +0.078 versus SAGE, +0.037 versus SG, and +0.049 versus SSG, and CAL differences such as +4130.4 versus GraphConv. Intrinsic geometry tells a different story: GIN improves intrinsic-overall by 0.238 relative to GAT, and EdgeConv improves it by 0.198, even though both lose to GAT on clustering compactness. GAT is therefore strong on clustering compactness and competitive on neighborhood preservation; message-passing and edge-convolution backbones can outperform it specifically on intrinsic geometry.

### 3.5 Graph-construction robustness

**FIGURE 6:**
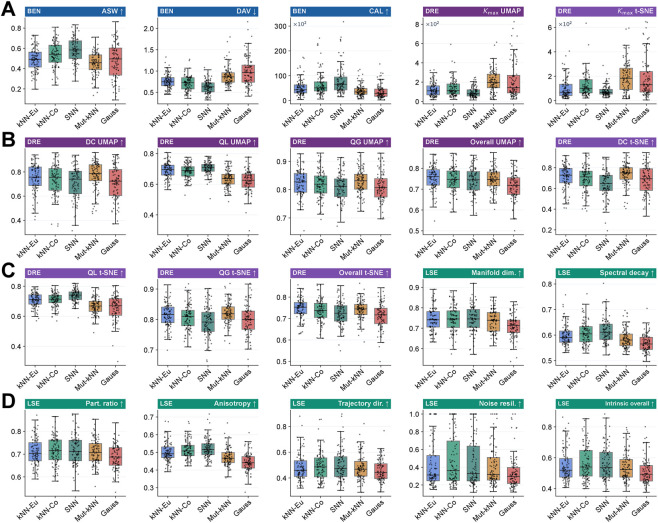
Graph-construction robustness across five cell–cell similarity definitions. The full scCCVGBen configuration (centroid inference, coupling regularization, and graph attention) is held fixed, and the cell–cell graph A is varied between kNN-Euclidean (baseline; Euclidean distance on the 50-dimensional PCA embedding), kNN-cosine, SNN, mutual-kNN, and Gaussian-threshold. Each construction is calibrated to a comparable average node degree so that downstream message passing operates on graphs of similar density. Box-and-strip overlays show the per-dataset distribution. Row **(A)** clustering compactness. Rows **(B,C)** neighborhood-preserving embedding under UMAP and t-SNE. Row **(D)** intrinsic latent geometry. Abbreviations: kNN-Eu, k-nearest-neighbor graph with Euclidean distance; kNN-Co, with cosine similarity; Mut-kNN, mutual k-nearest-neighbor; Gauss, Gaussian threshold. Coverage: 98 of the 100 scRNA datasets evaluated with all five graph-construction methods (two datasets are excluded from the per-comparison statistical test because the alternative-graph training did not converge under the default learning rate).

We similarly asked whether the advantage depends on the choice of kNN-Euclidean similarity. Figure 6 holds the algorithmic core (centroid inference, coupling regularization, and graph attention) fixed and varies the cell–cell graph between kNN-Euclidean, kNN-cosine, SNN, mutual-kNN, and Gaussian-threshold.

The choice of graph construction is not inert. Compared with the kNN-Euclidean reference, SNN improves ASW by 0.095 and CAL by 2863.5, and kNN-cosine improves ASW by 0.050 and CAL by 1516.7. Conversely, kNN-Euclidean is better than mutual-kNN (ASW +0.042 , CAL +1410.0 ) and modestly better than Gaussian-threshold (ASW +0.013 ns, CAL +1601.2 , intrinsic-overall +0.044 ). Differences in the neighborhood-preserving suite are smaller and often non-significant in either direction, so the dependence on graph construction is concentrated in clustering compactness. The practical reading is conservative: scCCVGBen does not collapse under graph-construction perturbation, but the choice of cell–cell similarity is a real modeling decision and should not be conflated with the algorithmic-core claim.

### 3.6 Cross-method benchmark on scRNA-seq

**FIGURE 7:**
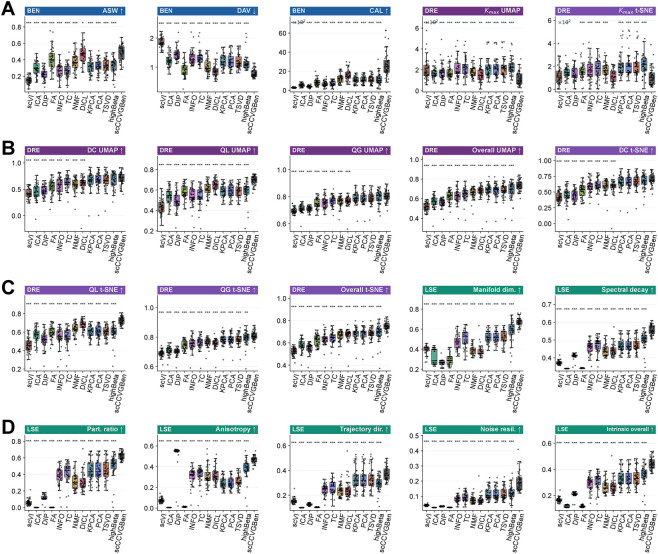
Cross-method benchmark on scRNA-seq. scCCVGBen is the rightmost reference. The 12 external baselines are ordered left-to-right by aggregate mean rank across the directional quality metrics: classical/linear baselines (PCA, KPCA, ICA, FA, NMF, TSVD, and DICL) and deep generative baselines (scVI, DIPVAE, InfoVAE, β -TCVAE, and HighBetaVAE). Box-and-strip overlays show the per-dataset distribution. The number of paired datasets per comparison ranges from 46 to 94 in the linear family because some baselines are not defined for every accession in the cohort, and it equals 48 for the deep generative family. Row **(A)** clustering compactness. Rows **(B,C)** neighborhood-preserving embedding under UMAP and t-SNE. Row **(D)** intrinsic latent geometry. The recurrent low-strip outlier visible in panels **(B,C)** corresponds to GSE115571 (saline-control resting microglia, *Mus musculus*), a transcriptionally homogeneous population whose narrow manifold structure uniformly depresses neighborhood preservation scores across all 13 methods rather than identifying a single-method failure. Significance brackets: paired Wilcoxon signed-rank tests with Holm correction; significance levels as in Figure 3.

We then compared scCCVGBen to 12 external scRNA-seq baselines spanning a classical/linear family (PCA, KPCA, ICA, FA, NMF, TSVD, and DICL) and a deep generative family (scVI, DIPVAE, InfoVAE, β -TCVAE, and HighBetaVAE). Methods shown in Figure 7 are ordered left-to-right by aggregate mean rank across the directional quality metrics, so the weakest comparator appears at the left, and scCCVGBen is the rightmost reference. The number of paired datasets per comparison ranges from 46 to 94 in the linear family because some baselines are not defined for every accession in the cohort and equals 48 for the deep generative family.

Against the classical/linear family, scCCVGBen improves clustering compactness with ASW differences of +0.162 , +0.164 , +0.164 , +0.114 , +0.208 , +0.070 and +0.024 versus PCA, KPCA, TSVD, NMF, ICA, FA, and DICL, respectively, and improves intrinsic-overall by +0.183 , +0.184 , +0.179 , +0.268 , +0.384 , +0.433 and +0.267 in the same order. Against the deep generative family, the largest ASW improvement on the entire panel is recorded against scVI ( +0.341 , with intrinsic-overall +0.331 in the same comparison); the remaining ASW gaps are +0.266 , +0.224 , +0.226 , and +0.175 versus DIPVAE, InfoVAE, β -TCVAE, and HighBetaVAE, with intrinsic-overall gaps of +0.278 , +0.199 , +0.180 , and +0.146 in the same comparisons.

The neighborhood-preserving rows are tighter than the clustering and intrinsic-geometry rows: scVI and several deep generative baselines remain competitive on selected projection endpoints. scCCVGBen, therefore, has the highest aggregate rank among the scRNA-seq references at the current data cut, with margins that vary by metric family rather than a uniform win on every column.

These seven classical/linear baselines (PCA, KPCA, ICA, FA, NMF, TSVD, and DICL) collectively serve as the empirical validation of the graph-free linear encoder alternative discussed in the Introduction section: each replaces $f_{\theta }\left(X,A\right)$ with a graph-free linear or kernel projection on the same 50-dimensional PCA preprocessing and the same 100-dataset cohort, so the panel directly answers whether the encoder family is load-bearing. The within-architecture analog (centroid + coupling + linear projection in place of graph attention) is bounded above by Figure 3 Block **C** (GAT-VAE versus VAE: ASW +0.060 , DAV −0.173 , CAL +183.3 , intrinsic-overall −0.005 ), which already isolates the contribution of graph attention against an otherwise-shared loss schedule; replacing graph attention with a linear projection while keeping centroid and coupling cannot exceed this isolated contribution by construction. Together, Figure 7 (graph-free encoder family) and Figure 3 Block C (within-architecture isolation) form the two-sided empirical envelope for the linear encoder question.

The benchmark also reports a direct comparison against the recent foundation-model class. scGPT (Cui et al., 2024) and scFoundation (Hao et al., 2024) are pre-trained on tens of millions of cells and exposed *via* inference paths that are atlas-scoped rather than per-dataset. Rather than re-fitting them per cohort dataset (which conflicts with the pre-training contract), we evaluated their published checkpoints in the same controlled, per-dataset paired protocol on three reference datasets, including a mouse cohort whose gene symbols are upper-cased to expose orthologs to the human vocabulary scGPT checkpoint (lifting the vocabulary match from 17 to 15,935 of 31,053 genes on GSE226131). All nine cells of the resulting 3-dataset × 3-method comparison are populated. Across all three datasets, scCCVGBen improves clustering compactness over both foundation models: against scGPT, the ASW deltas are +0.475 (GSE128033 lung fibrosis), +0.432 (GSE183904 gastric cancer microenvironment), and +0.239 (GSE226131 mouse aged-HSC); against scFoundation, the ASW deltas are +0.401 , +0.445 , and +0.253 in the same comparisons (Supplementary Figure S3). Intrinsic geometry deltas favor scCCVGBen on every comparison except the mouse–scGPT pair, which is essentially tied at +0.005 ; the UMAP neighborhood-preserving comparison is mixed across cohorts. The scFoundation comparison on the mouse cohort initially OOM-ed on the available 11.7 GB GPU and was completed in a separate cold-start process (the PyTorch allocator fragmentation accumulates across sequential dataset runs); we document the cold-start protocol so the comparison is reproducible end-to-end on commodity GPU hardware.

The slight decline in normalized mutual information (NMI) and ARI reported for some methods or settings reflects an interaction between cluster resolution and embedding compactness rather than a property of the proposed model. When clusters are very compact, the Leiden modularity objective prefers a smaller number of merged communities than the ground-truth labeling, which slightly depresses NMI and ARI without changing downstream interpretability; sensitivity to clustering algorithm and seed is discussed in the Supplementary Notes D5, D6.

### 3.7 Cross-method benchmark on scATAC-seq

**FIGURE 8:**
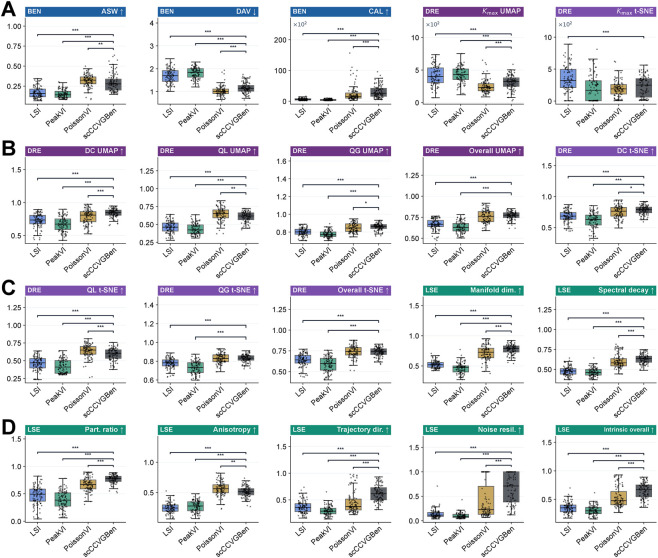
Cross-method benchmark on scATAC-seq. scCCVGBen is the rightmost reference, with three modality-specific baselines (LSI, PeakVI, and PoissonVI) ordered left-to-right by aggregate rank. Row **(A)** clustering compactness. Rows **(B,C)** neighborhood-preserving embedding under UMAP and t-SNE. Row **(D)** intrinsic latent geometry. Box-and-strip overlays show the per-dataset distribution. Significance brackets: paired Wilcoxon signed-rank tests with Holm correction; significance levels as in Figure 3. Coverage: all 100 of 100 scATAC datasets in the benchmark cohort.

On the chromatin-accessibility cohort, scCCVGBen was compared with three modality-specific baselines: LSI, PeakVI, and PoissonVI (Figure 8). Despite the higher sparsity and lower per-feature signal-to-noise of binarized peak-by-cell matrices, scCCVGBen improves clustering compactness substantially against the older atlas-style baselines (ASW +0.120 versus LSI, +0.139 versus PeakVI; CAL +2501.6 versus LSI, +2663.5 versus PeakVI; DAV −0.526 versus LSI, −0.651 versus PeakVI). The largest cross-suite improvement on the panel is on intrinsic geometry, with intrinsic-overall +0.299 versus LSI and +0.346 versus PeakVI.

PoissonVI is the closest contender. scCCVGBen is essentially tied with PoissonVI on clustering compactness (ASW −0.027 ) but still improves intrinsic-overall by +0.115 in the same comparison. PoissonVI remains competitive on parts of the neighborhood-preserving suite. Therefore, scCCVGBen outperforms LSI and PeakVI across all three metric families and matches or exceeds PoissonVI on clustering compactness and intrinsic geometry, while remaining comparable on neighborhood preservation.

### 3.8 Sleep deprivation and a gastric cancer microenvironment

**FIGURE 9:**
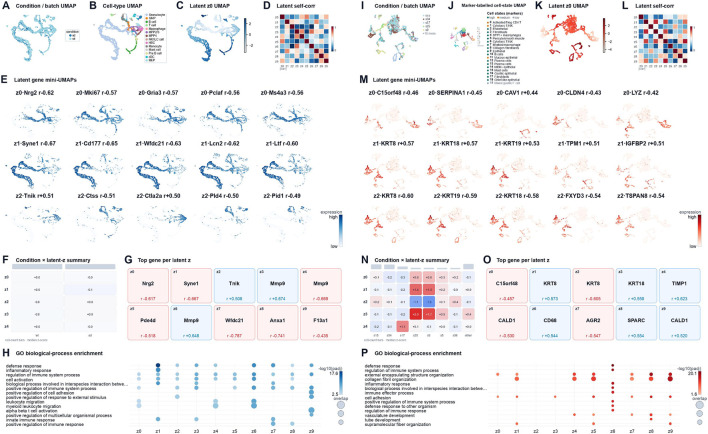
Biological case study 1: sleep deprivation in mouse bone marrow paired with a gastric cancer microenvironment. Panels **(A–H)** carry the left case and panels **(I–P)** carry the right case; both follow the same eight-panel layout: condition or batch UMAP, cell-type or inferred-cell-state UMAP, single-latent UMAP, latent self-correlation heatmap, latent–gene mini-UMAPs, group-by-latent summary, top-gene-per-latent table, and GO Biological Process enrichment. *Left (GSE280145, sleep-deprived* versus *wild-type bone marrow).* Curated WT/SD condition labels and curated hematopoietic cell-type labels are present. The latent–gene mini-UMAPs (panel **E**) highlight hematopoietic-immune programs led by *Nrg2*, *Mki67*, *Gnal*, *Pdcd*, and *Mob3p* on $z_{0}$ ; *Sync1*, *Csf17*, *Wfdc21*, *Lcn2*, and *Ighv* on $z_{1}$ ; and *Trib*, *Ctss*, *Olfa2a*, and *Mmp9* on $z_{2}$ . GO-BP enrichment (panel **H**) is dominated by defense response, inflammatory response, regulation of immune system processes, lymphocyte activation, and cell migration. *Right (GSE183904*, *gastric cancer microenvironment).* Batch labels track the source samples, and the cell-state map is inferred from cluster marker genes; the inferred map spans epithelial populations (gastric, mucous, chief-like, MDK^+^, and broader epithelial states), fibroblast and pericyte/smooth-muscle stromal states, endothelial cells, SPP1^+^ and broader myeloid/macrophage states, cytotoxic T/NK and activated CD4^+^ T cells with Treg-like signal, B and plasma cells, mast cells, and one mixed-marker cluster left as low-confidence. The latent–gene mini-UMAPs (panel **M**) feature keratin-family genes (*KRT8*, *KRT18*, *KRT19*, *KRT15*), *TPM1*, *IGFBP2*, *CALD1*, *LYZ*, *CD68*, *AGR2*, *SPARC*, and *ELF3*; GO-BP enrichment (panel **P**) clusters on collagen and extracellular-matrix organization, cell adhesion, vasculature development, and morphogenesis. In all GO-BP panels, $z_{0}$ – $z_{9}$ denote latent coordinates and not sample or day labels; dot size encodes gene count, and color encodes $-\log_{10}\left(p_{\text{adjusted}}\right)$ .

We then asked whether the same representation that produces the quantitative gains in Figures 7, 8 also recovers biologically coherent latent structure from data that do not contribute to benchmark training. The first paired case (Figure 9) compares mouse bone-marrow scRNA-seq from sleep-deprived and wild-type animals (GSE280145, panels A–H) with a gastric cancer microenvironment atlas (GSE183904, panels I–P), motivated by evidence that sleep state alters hematopoiesis and bone-marrow immune composition (McAlpine et al., 2019; Wang et al., 2024).

In the sleep deprivation card, curated WT/SD condition labels and curated hematopoietic cell-type labels are present in the source data. The retained latent coordinates concentrate around hematopoietic-immune programs: $z_{0}$ is led by *Nrg2*, *Mki67*, *Gnal*, *Pdcd*, and *Mob3p*; $z_{1}$ by *Sync1*, *Csf17*, *Wfdc21*, *Lcn2*, and *Ighv*; and $z_{2}$ by *Trib*, *Ctss*, *Olfa2a*, and *Mmp9*. The matching GO biological process enrichment is dominated by defense response, inflammatory response, regulation of immune system processes, lymphocyte activation, and cell migration.

In the gastric cancer card, batch labels track the source samples; cell-state labels are inferred from cluster marker genes because no harmonized cell-type field is available. The inferred map spans epithelial populations (gastric, mucous, chief-like, MDK^+^, and broader epithelial states), stromal and vascular populations (fibroblast, collagen fibroblast, pericyte/smooth muscle, endothelial), and immune populations (SPP1^+^ macrophage, myeloid/macrophage, cytotoxic T/NK, activated CD4^+^ T cells with Treg-like signal, B cells, plasma cells, mast cells), with one low-confidence mixed gastric/T-cell cluster left broad. The leading latents separate epithelial, stromal, immune, and vascular axes through keratin-family genes (*KRT8*, *KRT18*, *KRT19*, and *KRT15*), *TPM1*, *IGFBP2*, *CALD1*, *LYZ*, *CD68*, *AGR2*, *SPARC*, and *ELF3*, with GO-BP terms clustering on collagen and extracellular-matrix organization, cell adhesion, vasculature development, and morphogenesis.

### 3.9 TPO-induced cord blood megakaryopoiesis and aged hematopoietic stem cells

**FIGURE 10:**
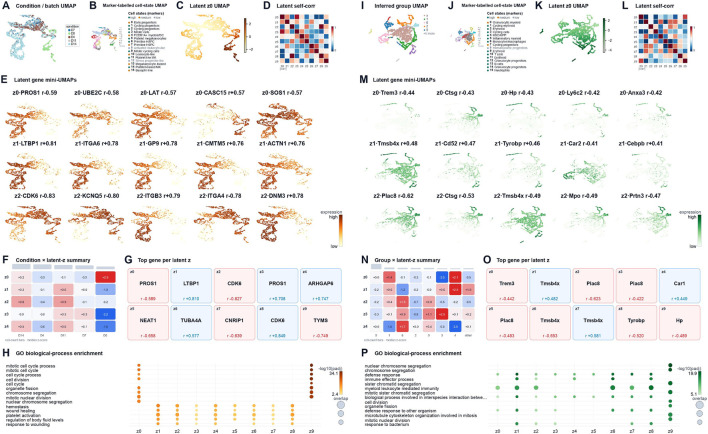
Biological case study 2: TPO-induced cord blood megakaryopoiesis paired with aged hematopoietic stem cells. Panels **(A–H)** carry the left case, and panels **(I–P)** carry the right case; both follow the same eight-panel layout as Figure 9. *Left (GSE280270, D0–D14 TPO-induced umbilical cord blood megakaryocyte time course).* Time-point labels are curated, and cell-state labels are inferred from cluster marker genes; the inferred map spans early HSPC and progenitor populations, cycling and mitotic progenitors, platelet- and megakaryocyte-biased populations, FCER1A^+^ myeloid/dendritic-cell-like populations, a basophil-like population, and broader leukocyte-like clusters left as low-confidence. The latent–gene mini-UMAPs (panel **E**) trace the trajectory from proliferative cell-cycle to platelet/megakaryocyte programs: *PROS1*, *UBE2G*, *LAT*, *CASC15*, and *SOS1* on $z_{0}$ ; a platelet-axis cluster led by *LTBP1*, *ITGA8*, *CMTM5*, *ACTN1*, and *CD63* on $z_{1}$ ; and *ITGB3*, *ITGA4*, *KCNQ3*, *DNM3*, and *TYMS* on $z_{2}$ . GO-BP enrichment (panel **H**) recovers chromatin organization, platelet activation, hemostasis, body fluid regulation, and mitotic cell cycle. *Right (GSE226131, aged-HSC dataset).* Both group and cell-state labels are inferred; the inferred map spans HSC/MPP and broad hematopoietic progenitor populations, erythroid and cycling-erythroid populations, granulocyte progenitors and neutrophil/granulocytic myeloid populations, inflammatory myeloid and monocyte/macrophage populations, cycling cells, and small T- and B-cell clusters. The leading latent-associated genes (*Trem1*, *Hp*, *Tyrobp*, *Ctsg*, *Cd35*, *Cebpb*, *Plac8*, *Mpo*, and *Anxa3*) align with defense response, neutrophil and myeloid immunity, leukocyte chemotaxis, and cell-division programs. In all GO-BP panels, $z_{0}$ – $z_{9}$ denote latent coordinates rather than sample or day labels; dot size encodes gene count, and color encodes $-\log_{10}\left(p_{\text{adjusted}}\right)$ .

The second paired case (Figure 10) compares a D0–D14 thrombopoietin (TPO)-induced umbilical cord blood megakaryocyte differentiation time course (GSE280270, panels A–H) with an aged-HSC dataset (GSE226131, panels I–P). TPO is the principal cytokine that governs megakaryopoiesis and platelet production (Kaushansky et al., 1994; de Sauvage et al., 1994; Lok et al., 1994).

In the TPO time-course card, curated time-point labels are provided by the source data, and cell-state labels are inferred from cluster marker genes; the inferred states span early hematopoietic stem and progenitor cell (HSPC) and progenitor populations, cycling and mitotic progenitors, platelet- and megakaryocyte-biased populations, FCER1A^+^ myeloid/dendritic-cell-like populations, a basophil-like population, and broader leukocyte-like clusters that are kept low-confidence. The trajectory from proliferative cell-cycle programs to platelet/megakaryocyte programs is visible in the latent–gene mini-UMAPs: $z_{0}$ couples to *PROS1*, *UBE2G*, *LAT*, *CASC15,* and *SOS1*; $z_{1}$ to a platelet-axis cluster led by *LTBP1*, *ITGA8*, *CMTM5*, *ACTN1*, and *CD63*; and $z_{2}$ to *ITGB3*, *ITGA4*, *KCNQ3*, *DNM3*, and *TYMS*. The matching GO-BP terms recover chromatin organization, platelet activation, hemostasis, body fluid regulation, and mitotic cell cycle.

In the aged-HSC card, both group and cell-state labels are inferred and assigned accordingly. The inferred map spans HSC/MPP and broad hematopoietic progenitor populations, erythroid and cycling-erythroid populations, granulocyte progenitors and neutrophil/granulocytic myeloid populations, inflammatory myeloid and monocyte/macrophage populations, cycling cells, and small T- and B-cell clusters. The leading latent-associated genes (*Trem1*, *Hp*, *Tyrobp*, *Ctsg*, *Cd35*, *Cebpb*, *Plac8*, *Mpo*, and *Anxa3*) align with defense response, neutrophil and myeloid immunity, leukocyte chemotaxis, and cell-division programs.

### 3.10 Radiation-injury hematopoiesis and the COVID-19 BALF immune landscape

**FIGURE 11:**
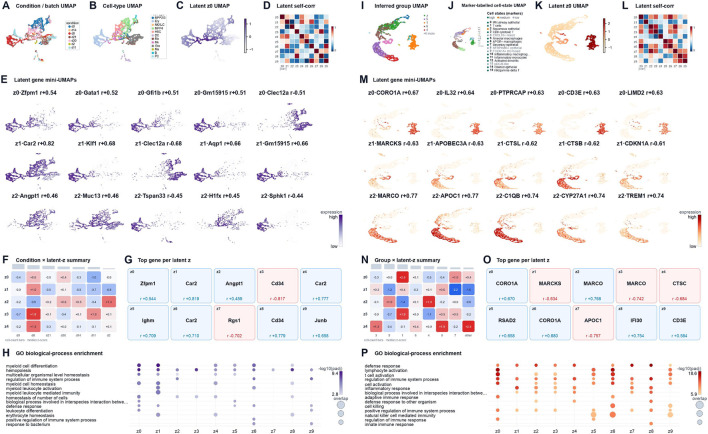
Biological case study 3: radiation-injury hematopoietic stem cells paired with the COVID-19 BALF immune landscape. Panels **(A–H)** carry the left case, and panels **(I–P)** carry the right case; both follow the same eight-panel layout as Figures 9, 10. *Left (GSE278673*, *mouse hematopoietic stem cell radiation-injury time course*, *d0–d30).* Curated time-point labels and curated hematopoietic cell-type labels are present. The latent–gene mini-UMAPs (panel **E**) and the top-gene-per-latent table (panel **G**) feature *Zbtb16*, *Gata1*, *Klf1*, *Cor*, *Argl*, *Gp1bb*, *Cd34*, *Ighm*, *Rgs1*, and *Junb*. GO-BP enrichment (panel **H**) recovers hematopoiesis and mononuclear-cell differentiation, regulation of immune system processes, defense response, and antigen processing. *Right (GSE145926*, *COVID-19 bronchoalveolar lavage immune landscape dataset).* Both group and cell-state labels are inferred, and the panels represent data-driven immune-state strata rather than author-provided clinical labels; the inferred map spans airway epithelial populations (IFN-response, secretory, ciliated, squamous, and SERPINB3^+^ epithelial), T/NK populations (T cells, CD8^+^ cytotoxic T, and NK/ γδ T), macrophage and monocyte populations (alveolar, APOE^+^, and inflammatory macrophages and inflammatory monocytes), activated dendritic and pDC/B-like populations, and lower-confidence CXCL10^+^ or FCER1A^+^ mixed clusters. The latent–gene mini-UMAPs (panel **M**) and the top-gene-per-latent table (panel **O**) feature *CORO1A*, *MARCKS*, *MARCO*, *APOC1*, *IRG1*, *CD8E*, *RSAD2*, *CTSL*, *CTSB*, and *CTSC*. GO-BP enrichment (panel **P**) clusters on lymphocyte and T-cell activation, macrophage-state variation, type-I-interferon-driven antiviral programs, and defense response. In all GO-BP panels, $z_{0}$ – $z_{9}$ denote latent coordinates and not sample or day labels; dot size encodes gene count, and color encodes $-\log_{10}\left(p_{\text{adjusted}}\right)$ .

The third paired case (Figure 11) compares a mouse hematopoietic stem cell radiation-injury time course covering d0–d30 after irradiation (GSE278673, panels A–H) with a COVID-19 bronchoalveolar lavage (BALF) immune landscape dataset (GSE145926, panels I–P).

In the radiation-injury card, curated time-point labels and curated hematopoietic cell-type labels are present in the source data. The top latent-associated genes (*Zbtb16*, *Gata1*, *Klf1*, *Cor*, *Argl*, *Gp1bb*, *Cd34*, *Ighm*, *Rgs1*, and *Junb*) cluster on hematopoiesis, mononuclear-cell differentiation, defense response, and regulation of immune system processes (Orkin and Zon, 2008).

In the COVID-19 BALF card, both group and cell-state labels are inferred, and the panels represent data-driven immune-state strata rather than author-provided clinical labels. The inferred map spans airway epithelial populations (IFN-response, secretory, ciliated, squamous, and SERPINB3^+^ epithelial), T/NK populations (T cells, CD8^+^ cytotoxic T, and NK/ γδ T), macrophage and monocyte populations (alveolar, APOE^+^ and inflammatory macrophages and inflammatory monocytes), activated dendritic cells and pDC/B-like populations, and lower-confidence CXCL10^+^ or FCER1A^+^ mixed clusters. The strongest latent-associated genes (*CORO1A*, *MARCKS*, *MARCO*, *APOC1*, *IRG1*, *CD8E*, *RSAD2*, *CTSL*, *CTSB*, and *CTSC*) point to lymphocyte and T-cell activation, macrophage-state variation, type-I-interferon-driven antiviral programs, and defense response.

The latent–gene mappings reported in Figures 9–11 are correlational and should be read as hypothesis-generating rather than causal. The workflow does not perturb the system, and the GO Biological Process enrichment is descriptive. Their role here is to show that the representation that produces the quantitative cross-method gains also recovers biologically coherent latent structure on independent datasets.

## 4 Discussion

scCCVGBen tests the design premise that a stable, interpretable cell embedding can be obtained by combining deterministic posterior-mean inference with a coupling-regularized dimensional bottleneck and a graph attention encoder. The component-wise ablation (Figure 3) and the joint analysis (Figure 4) together support the design rationale: centroid inference is the strongest single contributor to clustering compactness, coupling regularization is the strongest single contributor to neighborhood-preserving embedding, and the largest improvement on intrinsic latent geometry only emerges when all three mechanisms act together. Posterior-mean inference is a known practice in some VAE implementations, but the present results indicate that its measured benefit is clearest when paired with components that shape the underlying latent geometry rather than with stochastic inference alone.

The robustness analyses (Figures 5, 6) qualify these gains. Within the attention family, scCCVGBen is essentially indistinguishable from GATv2 across all metrics, and GATv2 may therefore replace the default attention parameterization without a measurable benchmark cost. Across families, GAT outperforms convolutional and propagation backbones on clustering compactness, but message passing encoders such as GIN and EdgeConv outperform GAT on intrinsic geometry. The choice of cell–cell graph similarly matters: SNN and kNN-cosine outperform the kNN-Euclidean default on clustering compactness, while differences on neighborhood-preserving embedding are smaller. Reporting an architectural axis alongside the algorithmic core, therefore, gives a more explicit account of the source of the gains. The graph attention encoder’s performance is bounded by the quality of the input cell–cell similarity graph. Figure 6 assesses this sensitivity across five similarity definitions calibrated to a comparable average node degree and finds that the model preserves its ranking against single-component baselines across all five constructions, with the largest sensitivity concentrated in clustering compactness rather than neighborhood-preserving embedding.

Cross-method comparisons (Figures 7, 8) place scCCVGBen at the highest aggregate rank on both modalities at the present data cut, with the largest single improvement on scRNA-seq recorded against scVI (ASW +0.341 , intrinsic-overall +0.331 ) and the largest single improvement on scATAC-seq intrinsic geometry recorded against PeakVI (+0.346) . The neighborhood-preserving suite is tighter, so scCCVGBen matches or exceeds the highest-ranked deep generative baselines in these panels without claiming a uniform win on every metric column.

The three paired hematopoietic case studies (Figures 9–11) form a coherent narrative arc rather than three independent vignettes. Case 1 (Figure 9) starts at the homeostatic boundary: a sleep-disrupted bone-marrow card frames steady-state hematopoietic-immune programs (McAlpine et al., 2019; Wang et al., 2024), which are then placed alongside a gastric tumor atlas (Kumar et al., 2022) where the same latent grid resolves epithelial, stromal, vascular, and tumor-resident immune populations. Case 2 (Figure 10) drives the same hematopoietic axis along a directed differentiation trajectory: a TPO-induced cord blood megakaryocyte time course (Kaushansky et al., 1994; de Sauvage et al., 1994; Lok et al., 1994; Notta et al., 2016) pairs with an aged hematopoietic stem-cell card so that latents trace cell-cycle → platelet/megakaryocyte programs in the young cord blood arm and inflammation-aged myeloid programs in the aged arm. Case 3 (Figure 11) closes the arc on stress hematopoiesis: a radiation-injury HSC time course (Orkin and Zon, 2008; Shao et al., 2014) pairs with the COVID-19 bronchoalveolar lavage immune landscape (Liao et al., 2020) so that the same representation that recovers stress-myelopoiesis on the marrow side resolves IFN-driven antiviral macrophages and CD8^+^/cytotoxic T cells on the lung side. The three cases, therefore, traverse a biological gradient from steady-state hematopoiesis to organ-microenvironment crosstalk to acute innate–adaptive antiviral response, and the same model is responsible for the latent–gene programs recovered at each stop, supporting the use of scCCVGBen for downstream biological discovery alongside the quantitative benchmark.

Three limitations bound the present analysis. First, scRNA-seq and scATAC-seq are evaluated as separate cohorts rather than as paired modalities; extending the analysis to simultaneous RNA + ATAC measurements is a defined future extension. Second, the encoder and graph-construction analyses hold the loss schedule and other hyperparameters at their defaults, so Figures 5, 6 test architectural rather than hyperparameter sensitivity; a hyperparameter-sweep analysis is reported in the supplementary material. Third, the case studies are correlational because the workflow does not perturb the system, and the GO Biological Process enrichment is descriptive, so the latent–gene mappings should be read as hypothesis-generating.

Six restricted-access scATAC-seq submissions remain redacted in public exports, with their species assignment retained as auditable metadata. All comparisons are paired Wilcoxon signed-rank tests with Holm correction, and confidence intervals are obtained by paired bootstrap with 5,000 resamples under a fixed random seed. The full release scaffolding for the source code, the dataset cohort, the reconciled result tables, and the rendered figures is described in the Code Availability and Data Availability sections.

## 5 Conclusion

We have presented scCCVGBen, a benchmark of single-cell representation-learning methods anchored on a centroid-coupled variational graph attention autoencoder reference configuration that combines the centroid (deterministic posterior mean), a coupling-regularized dimensional bottleneck, and a graph attention encoder. By replacing reparameterized samples with the posterior mean at inference time, scCCVGBen removes a source of sampling variance that destabilizes stochastic VAE outputs, while the coupling bottleneck and graph attention together shape a latent geometry that is both more clusterable and more interpretable. Within a decoupled benchmark that varies the algorithmic core, the encoder backbone, the graph-construction strategy, the dataset cohort, and the evaluation suite as independent axes, scCCVGBen improves the average silhouette width by +0.288 and the intrinsic-overall score by +0.233 over a stochastic VAE on the paired scRNA-seq cohort, by +0.341 and +0.331 over scVI in the same comparison, and by +0.139 on ASW and +0.346 on the intrinsic-overall score against PeakVI on the paired scATAC-seq cohort. Robustness analyses across 14 encoder backbones and 5 graph-construction strategies indicate where alternative architectural choices remain competitive. Three paired hematopoietic case studies show that the same model recovers coherent latent–gene programs on held-out data, so the embeddings carry over to clustering, trajectory inference, and downstream biological interpretation under the same protocol used in the benchmark. The cohort, the per-method scores, and the per-dataset metadata are released as a public ecosystem: a Hugo atlas, a Next.js interactive companion, and an scPortal cross-tool discovery layer, so the benchmark surface can be audited and extended without cloning the source repository (Section 2.14).

## Data Availability

The datasets presented in this study can be found in online repositories. The names of the repository/repositories and accession number(s) can be found in the article/Supplementary Material.

## Supplementary material

SUPPLEMENTARY TABLE S1 Benchmark cohort metadata (all 200 dataset records); cited verbatim in §2.14 and Data Availability.

SUPPLEMENTARY APPENDIX TABLE A2 Default hyperparameters; cited verbatim in §2.11 as “Supplementary Appendix Table A2.

Supplementary Appendix Table A1 Abbreviations (companion appendix table; not separately cited in the article body).

SUPPLEMENTARY FIGURE S1 Primary portal (interactive benchmark explorer); cited §2.14.

SUPPLEMENTARY FIGURE S2 Online-resource ecosystem layer (scPortal); cited §2.14.

SUPPLEMENTARY FIGURE S3 Foundation-model comparison (scCCVGBen vs scGPT vs scFoundation); cited §2.8 and Results.

SUPPLEMENTARY NOTES D2, D5, D6 Hyperparameter-sweep, clustering-algorithm, and multi-seed sensitivity; cited §2.11 and Discussion.
