# Supplementary material for: scCCVGBen for benchmarking of single-cell representation learning anchored on a centroid-coupled variational graph attention autoencoder across scRNA-seq and scATAC-seq
Source: Front Genet. 2026 Jun 16;17:1822168. doi: 10.3389/fgene.2026.1822168 (PMC13313601; doi:10.3389/fgene.2026.1822168)
Supplement: Supplementary file 1 [file Supplementaryfile1.pdf]

Supplementary Information for scCCVGBen for benchmarking of single-cell representation learning anchored on a centroid-coupled variational graph attention autoencoder across scRNA-seq and scATAC-seq

Zeyu Fu[1†], Jiawei Fu[2†], Chunlin Chen[3†], Keyang Zhang[4], Junping Wang[1*], Tianfei Ran[2*], Song Wang[1*]

[1]State Key Laboratory of Trauma and Chemical Poisoning, Institute of Combined Injury, Chongqing Engineering Research Center for Nanomedicine, College of Preventive Medicine, Army Medical University, Chongqing, 400038, China.
[2]Department of Orthopedics, Xinqiao Hospital, Army Medical University, Chongqing, 400037, China.
[3]Department of Rehabilitation Medicine, The First Affiliated Hospital, Sun Yat-sen University, Guangzhou, 510080, China.
[4]School of Medicine, Sun Yat-sen University, Shenzhen, 518107, China.

*Corresponding author(s). E-mail(s): wangjunping@tmmu.edu.cn; rantianfei@tmmu.edu.cn; swang1981@tmmu.edu.cn;
Contributing authors: fuzeyu99@126.com; fjw813130855@163.com; chenchlin3@mail2.sysu.edu.cn; zhangky39@mail2.sysu.edu.cn;
†These authors contributed equally to this work.

Abstract

This document is the Supplementary Information for the above article. It collects the table of abbreviations (Appendix Table A1), the full benchmark-cohort metadata table (Supplementary Table S1), the default-hyperparameter configuration (Appendix Table A2), the auxiliary online-resource figures and the foundation-model comparison figure (Supplementary Figs. S1–S3), and supplementary notes on hyperparameter and clustering-algorithm sensitivity (D2, D5, D6). Cross-references to "main-text" items point to the corresponding figures and sections of the primary article.

1 Abbreviations

Appendix Table A1: Abbreviations used throughout the main article and this Supplementary Information.

Abbreviation	Meaning
scCCVGBen	single-cell Centroid-Coupled Variational Graph autoencoder Benchmark
scRNA-seq	single-cell RNA sequencing
scATAC-seq	single-cell Assay for Transposase-Accessible Chromatin sequencing
GEO	Gene Expression Omnibus
ENA	European Nucleotide Archive
HVG	highly variable gene
PCA	Principal Component Analysis
LSI	Latent Semantic Indexing
TF–IDF	Term Frequency–Inverse Document Frequency

(continued on next page)

Abbreviations (continued)

Abbreviation	Meaning
VAE	Variational Autoencoder
VGAE	Variational Graph Autoencoder
CenVAE	centroid-inference-only ablation variant
CouVAE	coupling-regularisation-only ablation variant
GAT	Graph Attention Network
GATv2	second-generation Graph Attention Network
GCN	Graph Convolutional Network
SAGE	GraphSAGE inductive graph encoder
GraphConv	generic message-passing graph convolution
Cheb	Chebyshev spectral graph convolution
TAG	Topology Adaptive Graph convolution
ARMA	Auto-Regressive Moving-Average graph convolution
SG	Simple Graph convolution
SSG	Stacked Simple Graph convolution
GIN	Graph Isomorphism Network
EdgeConv	dynamic edge convolution
kNN	k-nearest-neighbour graph
SNN	Shared-Nearest-Neighbour graph
mutual-kNN	symmetric intersection of two kNN graphs
ASW	Average Silhouette Width (BEN suite)
DAV	Davies–Bouldin index (BEN suite)
CAL	Calinski–Harabasz index (BEN suite)
$K_{\max}$	maximum 2D cluster size in UMAP/t-SNE projection
DC	distance correlation against the high-dimensional input (DRE suite)
$Q_{\text{local}}, Q_{\text{global}}$	local and global co-ranking quality scores (DRE suite)
BEN	clustering compactness suite (3 metrics)
DRE	neighbourhood-preserving embedding suite (10 metrics)
LSE	intrinsic latent geometry suite (7 metrics)
UMAP	Uniform Manifold Approximation and Projection
t-SNE	t-distributed Stochastic Neighbour Embedding
GO-BP	Gene Ontology Biological Process enrichment
HSC	hematopoietic stem cell
MPP	multipotent progenitor
HSPC	hematopoietic stem and progenitor cell
TPO	thrombopoietin
UCB	umbilical cord blood
BALF	bronchoalveolar lavage fluid

2 Supplementary Material

This consolidated supplementary section contains the full dataset table (Supplementary Table S1), the default-hyperparameter configuration (Appendix Table A2), the two auxiliary online-resource figures (Supplementary Figs. S1 and S2) and the foundation-model comparison figure (Supplementary Fig. S3, scCCVGBen vs. scGPT and scFoundation on three reference datasets).

Supplementary Table S1: Benchmark cohort metadata. All 200 public dataset records are listed with their accession-safe dataset ID, modality, GEO/GSM accession, cell count, species, tissue annotation and GEO URL.

Dataset ID	Modality	Accession	Cells	Species	Tissue	Source
GSE115571	scRNA	GSE115571	19 998	mouse	brain	GEO link
GSE117988	scRNA	GSE117988	12 874	human	pbmc	GEO link
GSE120505	scRNA	GSE120505	14 588	mouse	blood	GEO link
GSE123813	scRNA	GSE123813	53 030	human	tumor	GEO link
GSE123902	scRNA	GSE123902	42 847	human	lung	GEO link
GSE124310	scRNA	GSE124310	27 796	human	tumor	GEO link
GSE130148	scRNA	GSE130148	10 360	human	lung	GEO link
GSE130646	scRNA	GSE130646	2876	human	muscle	GEO link
GSE132509	scRNA	GSE132509	39 375	human	pbmc	GEO link
GSE138709	scRNA	GSE138709	33 991	human	liver	GEO link
GSE142653	scRNA	GSE142653	5181	human	other	GEO link
GSE143423	scRNA	GSE143423	12 196	human	brain	GEO link

Continued on next page

Table S1 (continued)

Dataset ID	Modality	Accession	Cells	Species	Tissue	Source
GSE145929	scRNA	GSE145929	61 014	mouse	bladder	GEO link
GSE148215	scRNA	GSE148215	10 110	human	stem_cell	GEO link
GSE148218	scRNA	GSE148218	50 747	human	tumor	GEO link
GSE149655	scRNA	GSE149655	13 060	human	tumor	GEO link
GSE155109	scRNA	GSE155109	8433	human	tumor	GEO link
GSE165784	scRNA	GSE165784	11 492	human	retina	GEO link
GSE165844	scRNA	GSE165844	19 791	mouse	bone_marrow	GEO link
GSE167597	scRNA	GSE167597	16 042	mouse	brain	GEO link
GSE168181	scRNA	GSE168181	23 556	mouse	tumor	GEO link
GSE183904	scRNA	GSE183904	62 035	human	tumor	GEO link
GSE189070	scRNA	GSE189070	103 446	mouse	brain	GEO link
GSE189357	scRNA	GSE189357	45 938	human	lung	GEO link
GSE192857	scRNA	GSE192857	11 067	human	stem_cell	GEO link
GSE213740	scRNA	GSE213740	23 871	human	other	GEO link
GSE222002	scRNA	GSE222002	78 315	mouse	tumor	GEO link
GSE222369	scRNA	GSE222369	138 805	human	tumor	GEO link
GSE225600	scRNA	GSE225600	81 683	human	tumor	GEO link
GSE225857	scRNA	GSE225857	22 260	human	liver	GEO link
GSE226131	scRNA	GSE226131	15 793	mouse	bone_marrow	GEO link
GSE228499	scRNA	GSE228499	32 349	human	tumor	GEO link
GSE235787	scRNA	GSE235787	113 495	human	tumor	GEO link
GSE247719	scRNA	GSE247719	696 410	mouse	muscle	GEO link
GSE253355	scRNA	GSE253355	99 846	human	bone_marrow	GEO link
GSE255019	scRNA	GSE255019	22 602	mouse	bone_marrow	GEO link
GSE262288	scRNA	GSE262288	157 518	human	tumor	GEO link
GSE275119	scRNA	GSE275119	6764	mouse	other	GEO link
GSE283205	scRNA	GSE283205	16 506	human	tumor	GEO link
GSE98638	scRNA	GSE98638	5063	human	liver	GEO link
GSE120446	scRNA	GSE120446	90 653	human	bone_marrow	GEO link
GSE95753	scRNA	GSE95753	18 213	mouse	brain	GEO link
GSE132188	scRNA	GSE132188	2531	mouse	pancreas	GEO link
GSE84133	scRNA	GSE84133	8569	human	pancreas	GEO link
GSE144024	scRNA	GSE144024	9485	human	stem_cell	GEO link
GSE226824	scRNA	GSE226824	13 310	mouse	bone_marrow	GEO link
GSE141259	scRNA	GSE141259	24 882	mouse	lung	GEO link
ds-scrna-047	scRNA	PRJEB37166	5780	human	bone_marrow	GEO link
GSE185538	scRNA	GSE185538	24 573	rat	embryo	GEO link
GSE130430	scRNA	GSE130430	886	human	bone_marrow	GEO link
GSE155249	scRNA	GSE155249	3595	human	breast	GEO link
GSE205506	scRNA	GSE205506	1666	human	neuro	GEO link
GSE139324	scRNA	GSE139324	1725	human	hnscc	GEO link
GSE167118	scRNA	GSE167118	3273	human	brain	GEO link
GSE162454	scRNA	GSE162454	8894	human	liver	GEO link
GSE145926	scRNA	GSE145926	6249	human	balf	GEO link
GSE166635	scRNA	GSE166635	16 077	human	brain	GEO link
GSE163558	scRNA	GSE163558	2866	human	stomach	GEO link
GSE164378	scRNA	GSE164378	161 764	human	pbmc	GEO link
GSE149689	scRNA	GSE149689	85 144	human	blood	GEO link
GSE151530	scRNA	GSE151530	56 721	human	liver	GEO link
GSE158803	scRNA	GSE158803	1092	human	neural	GEO link
GSE163484	scRNA	GSE163484	8203	mouse	brain	GEO link
GSE175814	scRNA	GSE175814	6223	human	lung	GEO link
GSE181061	scRNA	GSE181061	108 421	human	kidney	GEO link
GSE186344	scRNA	GSE186344	2228	human	bladder	GEO link
GSE197177	scRNA	GSE197177	21 560	human	gut	GEO link
GSE213337	scRNA	GSE213337	8631	mouse	pancreas	GEO link
GSE235063	scRNA	GSE235063	6757	human	thymus	GEO link
GSE139555	scRNA	GSE139555	7462	human	tcell	GEO link
GSE149655-2	scRNA	GSE149655	4397	human	airway	GEO link
GSE154778	scRNA	GSE154778	585	human	pancreas	GEO link
GSE181388	scRNA	GSE181388	22 157	human	muscle	GEO link
GSE188676	scRNA	GSE188676	22 360	mouse	retina	GEO link
GSE125449	scRNA	GSE125449	5115	human	liver	GEO link
GSE128033	scRNA	GSE128033	737 280	human	immune	GEO link
GSE138794	scRNA	GSE138794	2888	human	brain	GEO link
GSE159519	scRNA	GSE159519	13 229	human	lung	GEO link
GSE161277	scRNA	GSE161277	2947	human	kidney	GEO link
GSE173193	scRNA	GSE173193	7829	human	adipose	GEO link
GSE190604	scRNA	GSE190604	103 805	human	ovary	GEO link
GSE120926	scRNA	GSE120926	102 525	human	tumor	GEO link
GSE189357-2	scRNA	GSE189357	15 216	human	colon	GEO link
GSE208074	scRNA	GSE208074	8048	human	brain	GEO link
GSE220297	scRNA	GSE220297	9721	pig	blood	GEO link
GSE220840	scRNA	GSE220840	4913	human	embryo	GEO link
GSE260778	scRNA	GSE260778	3842	human	lung	GEO link
GSE264586	scRNA	GSE264586	13 254	human	kidney	GEO link

Continued on next page

Table S1 (continued)

Dataset ID	Modality	Accession	Cells	Species	Tissue	Source
GSE270891	scRNA	GSE270891	8920	human	stem_cell	GEO link
GSE273423	scRNA	GSE273423	7226	human	blood	GEO link
GSE274905	scRNA	GSE274905	30 634	human	ovary	GEO link
GSE279264	scRNA	GSE279264	12 071	human	stem_cell	GEO link
GSE280258	scRNA	GSE280258	11 104	human	pbmc	GEO link
GSE280767	scRNA	GSE280767	11 645	human	pbmc	GEO link
GSE283554	scRNA	GSE283554	6119	human	pbmc	GEO link
GSE193807	scRNA	GSE193807	8647	human	skin	GEO link
GSE216673	scRNA	GSE216673	13 867	human	brain	GEO link
GSE232938	scRNA	GSE232938	13 070	human	stem_cell	GEO link
GSE253936	scRNA	GSE253936	3267	human	blood	GEO link
GSE272457	scRNA	GSE272457	8265	human	kidney	GEO link
GSM8685152	scATAC	GSM8685152	27 166	mouse	other	GEO link
GSM8685177	scATAC	GSM8685177	22 409	mouse	other	GEO link
GSM8900551	scATAC	GSM8900551	20 020	mouse	lung	GEO link
GSM8900548	scATAC	GSM8900548	20 005	mouse	lung	GEO link
GSM8397466	scATAC	GSM8397466	18 157	mouse	bone_marrow	GEO link
GSM8900549	scATAC	GSM8900549	16 778	mouse	lung	GEO link
GSM8685155	scATAC	GSM8685155	16 236	mouse	other	GEO link
GSM8852349	scATAC	GSM8852349	16 176	human	tumor	GEO link
GSM7734291	scATAC	GSM7734291	15 709	mouse	blood	GEO link
GSM8900550	scATAC	GSM8900550	15 701	mouse	lung	GEO link
GSM8900553	scATAC	GSM8900553	14 346	mouse	lung	GEO link
GSM7734292	scATAC	GSM7734292	13 313	mouse	blood	GEO link
GSM7734290	scATAC	GSM7734290	12 883	mouse	blood	GEO link
GSM7308367	scATAC	GSM7308367	12 773	human	blood	GEO link
GSM8546125	scATAC	GSM8546125	10 772	mouse	lung	GEO link
GSM7064215	scATAC	GSM7064215	10 545	human	retina	GEO link
GSM7884541	scATAC	GSM7884541	9868	human	blood	GEO link
GSM8852348	scATAC	GSM8852348	9865	human	tumor	GEO link
GSM7884543	scATAC	GSM7884543	9640	human	retina	GEO link
GSM8546124	scATAC	GSM8546124	9531	mouse	lung	GEO link
GSM5402770	scATAC	GSM5402770	9495	mouse	blood	GEO link
GSM8997246	scATAC	GSM8997246	9346	human	blood	GEO link
GSM7308369	scATAC	GSM7308369	9302	human	blood	GEO link
GSM7884549	scATAC	GSM7884549	9041	human	blood	GEO link
GSM7064216	scATAC	GSM7064216	8905	human	retina	GEO link
GSM8462143	scATAC	GSM8462143	8872	human	lung	GEO link
GSM8462145	scATAC	GSM8462145	8389	human	lung	GEO link
GSM6214535	scATAC	GSM6214535	8253	mouse	gut	GEO link
GSM6052781	scATAC	GSM6052781	8016	mouse	skin	GEO link
GSM6638254	scATAC	GSM6638254	7990	human	other	GEO link
GSM8729707	scATAC	GSM8729707	7934	human	other	GEO link
GSM6052783	scATAC	GSM6052783	7896	mouse	skin	GEO link
GSM6052782	scATAC	GSM6052782	7714	mouse	skin	GEO link
GSM7884545	scATAC	GSM7884545	7682	human	blood	GEO link
GSM7064217	scATAC	GSM7064217	7102	human	retina	GEO link
GSM7734712	scATAC	GSM7734712	7028	human	other	GEO link
GSM7777063	scATAC	GSM7777063	6819	human	blood	GEO link
GSM7056609	scATAC	GSM7056609	6654	mouse	other	GEO link
GSM6044075	scATAC	GSM6044075	6617	human	liver	GEO link
GSM7777062	scATAC	GSM7777062	6433	human	blood	GEO link
GSM7884550	scATAC	GSM7884550	6390	human	blood	GEO link
GSM7734715	scATAC	GSM7734715	6357	human	other	GEO link
GSM7884540	scATAC	GSM7884540	5876	human	blood	GEO link
GSM7777060	scATAC	GSM7777060	5814	human	blood	GEO link
GSM7062447	scATAC	GSM7062447	5764	mouse	blood	GEO link
GSM7734716	scATAC	GSM7734716	5754	human	other	GEO link
GSM5769461	scATAC	GSM5769461	5409	zebrafish	other	GEO link
GSM6044077	scATAC	GSM6044077	5280	human	liver	GEO link
GSM5769453	scATAC	GSM5769453	5264	zebrafish	other	GEO link
GSM7062446	scATAC	GSM7062446	5171	mouse	blood	GEO link
GSM8462147	scATAC	GSM8462147	5048	human	lung	GEO link
GSE198730	scATAC	GSE198730	5010	mouse	stem_cell	GEO link
GSE198730-2	scATAC	GSE198730	5000	mouse	stem_cell	GEO link
GSM5766892	scATAC	GSM5766892	4901	human	stem_cell	GEO link
GSM5402771	scATAC	GSM5402771	4745	human	blood	GEO link
GSM8517035	scATAC	GSM8517035	4425	mouse	brain	GEO link
GSM7062449	scATAC	GSM7062449	4379	mouse	blood	GEO link
GSM5769456	scATAC	GSM5769456	4320	zebrafish	other	GEO link
GSM8397467	scATAC	GSM8397467	4207	mouse	bone_marrow	GEO link
GSM8462144	scATAC	GSM8462144	4116	human	lung	GEO link
GSM7064214	scATAC	GSM7064214	4112	human	retina	GEO link
GSM7777061	scATAC	GSM7777061	4074	human	blood	GEO link
GSM5975166	scATAC	GSM5975166	4007	mouse	other	GEO link
GSM5975165	scATAC	GSM5975165	3805	mouse	other	GEO link

Continued on next page

Table S1 (continued)

Dataset ID	Modality	Accession	Cells	Species	Tissue	Source
GSM6783752	scATAC	GSM6783752	3722	human	other	GEO link
GSM7062450	scATAC	GSM7062450	3693	mouse	blood	GEO link
GSM6449881	scATAC	GSM6449881	3473	human	blood	GEO link
GSM5124061	scATAC	GSM5124061	3350	human	pdx	GEO link
GSM6044076	scATAC	GSM6044076	3329	human	liver	GEO link
GSM6449880	scATAC	GSM6449880	3303	human	pbmc	GEO link
GSM6044073	scATAC	GSM6044073	3268	human	tumor	GEO link
GSM5769462	scATAC	GSM5769462	3267	zebrafish	other	GEO link
GSM5769463	scATAC	GSM5769463	3187	zebrafish	other	GEO link
GSM6449878	scATAC	GSM6449878	3179	human	other	GEO link
GSM8852347	scATAC	GSM8852347	3147	human	tumor	GEO link
GSM6783751	scATAC	GSM6783751	3084	human	other	GEO link
GSE252523	scATAC	GSE252523	4446	human	brain	GEO link
GSE270148	scATAC	GSE270148	4867	human	immune	GEO link
GSE171599	scATAC	GSE171599	6171	human	other	GEO link
restricted-scatac-001	scATAC	restricted	3134	human	blood	
restricted-scatac-002	scATAC	restricted	4196	human	blood	
restricted-scatac-003	scATAC	restricted	6718	human	blood	
restricted-scatac-004	scATAC	restricted	4802	human	blood	
restricted-scatac-005	scATAC	restricted	3020	human	blood	
GSE299741	scATAC	GSE299741	4805	mouse	other	GEO link
restricted-scatac-006	scATAC	restricted	3361	human	blood	
GSE272181	scATAC	GSE272181	7188	mouse	brain	GEO link
GSE288015	scATAC	GSE288015	4865	mouse	immune	GEO link
GSE289708	scATAC	GSE289708	6604	mouse	immune	GEO link
GSE295436	scATAC	GSE295436	4723	human	immune	GEO link
GSE299466	scATAC	GSE299466	13 347	mouse	immune	GEO link
GSE299558	scATAC	GSE299558	8934	mouse	lung	GEO link
GSE237304	scATAC	GSE237304	6824	mouse	other	GEO link
GSE301287	scATAC	GSE301287	5641	human	stem_cell	GEO link
GSE248713	scATAC	GSE248713	6849	mouse	other	GEO link
GSE214082	scATAC	GSE214082	11 054	mouse	other	GEO link
GSE234574	scATAC	GSE234574	7722	mouse	immune	GEO link
GSE201793	scATAC	GSE201793	8404	human	stem_cell	GEO link
GSE213469	scATAC	GSE213469	4971	mouse	immune	GEO link
GSE216175	scATAC	GSE216175	86 044	human	bone_marrow	GEO link

Default hyperparameters

Appendix Table A2: Default hyperparameters for the scCCVGBen configuration. Values are held fixed across the cohort and across the fourteen evaluated encoder backbones; figures that vary an architectural axis keep every other entry in this table unchanged.

Category	Parameter	Value
Architecture	Latent dimension (d_z)	10
	Bottleneck dimension (d_c)	5
	Hidden dimension	128
	Encoder hidden layers	2
	Decoder hidden dimension	128
	GAT attention heads	4
	GAT layers	2
	Dropout	0.05
Loss weights	w_{recon}	1.0
	w_{irecon}	1.0
	w_{KL}	1.0
	w_{adj}	1.0
Training	Learning rate	1×10^{-4}
	Optimizer	Adam
	Subgraph size (N_s)	512
	Subgraphs per epoch	10
	Training epochs	300
Graph construction	k-NN neighbours	15
	PCA dimensions	50

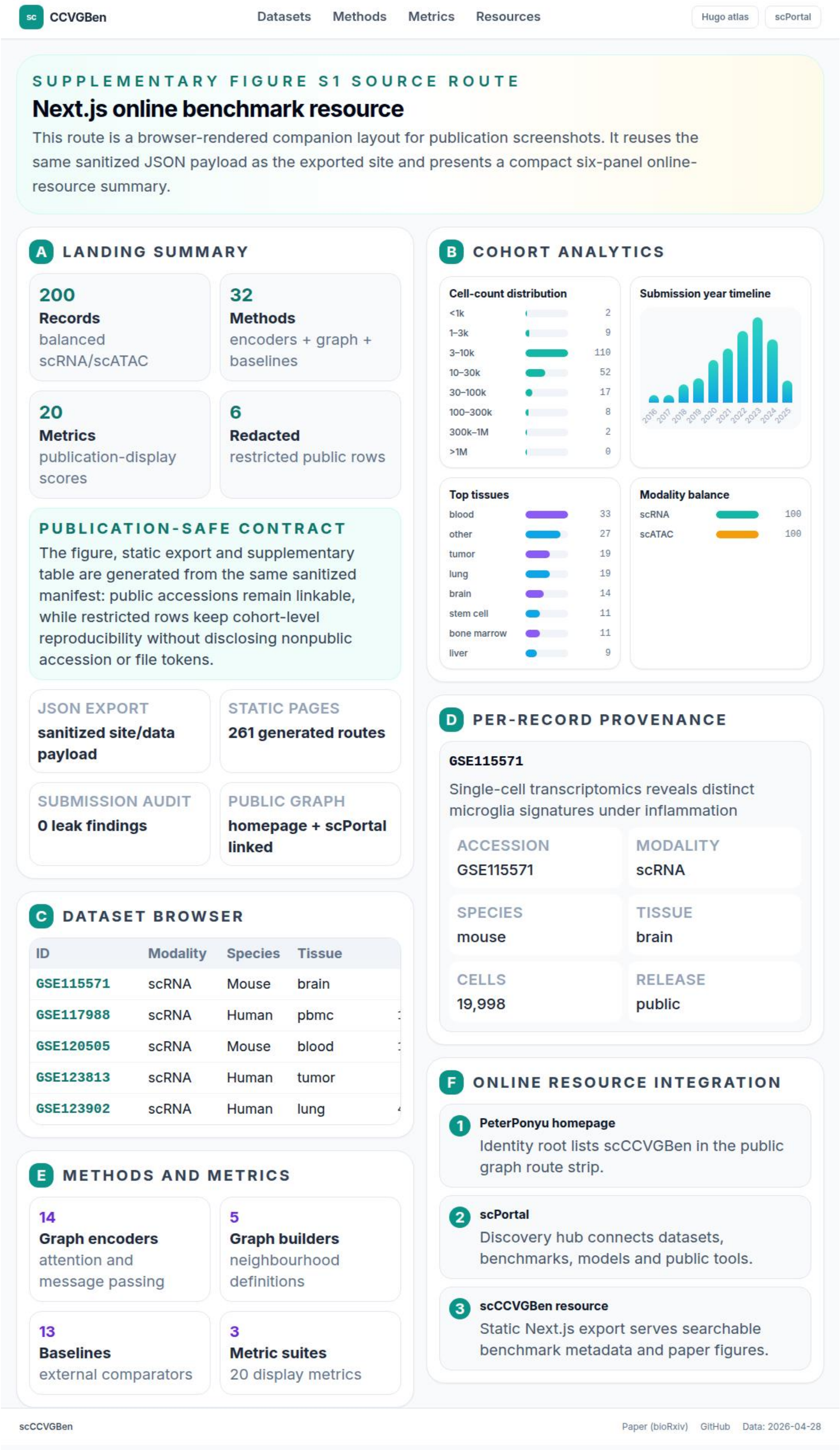

Fig. S1 scCCVGBen primary portal — Next.js interactive benchmark explorer. A publication-scale rendering of the project's primary web frontend, which combines a Hugo atlas at https://peterponyu.github.io/scCCVGBen/ with a Next.js interactive companion at https://peterponyu.github.io/scccvgben-next/. **(A)** Landing summary with the benchmark counts and primary-portal entry points. **(B)** Cohort composition charts for modality, species and submission year. **(C)** Dataset browser listing accession-safe public records with modality, species, tissue and cell-count columns. **(D)** Per-record metadata view of a public benchmark accession (example: GSE115571) showing the accession-safe fields exported by the manifest; the count of restricted-access rows omitted from this view is reported in panel A under "Redacted". **(E)** Method and metric taxonomy cards for the 32 benchmark methods and 20 publication-display metrics. **(F)** Online-resource integration panel showing how the scCCVGBen primary portal links out to its ecosystem context (scPortal); the broader ecosystem layer is documented in the next supplementary figure. The figure is composed by the project figure script from the same sanitized JSON data that drives the static export, avoiding manual LaTeX subpanel placement.

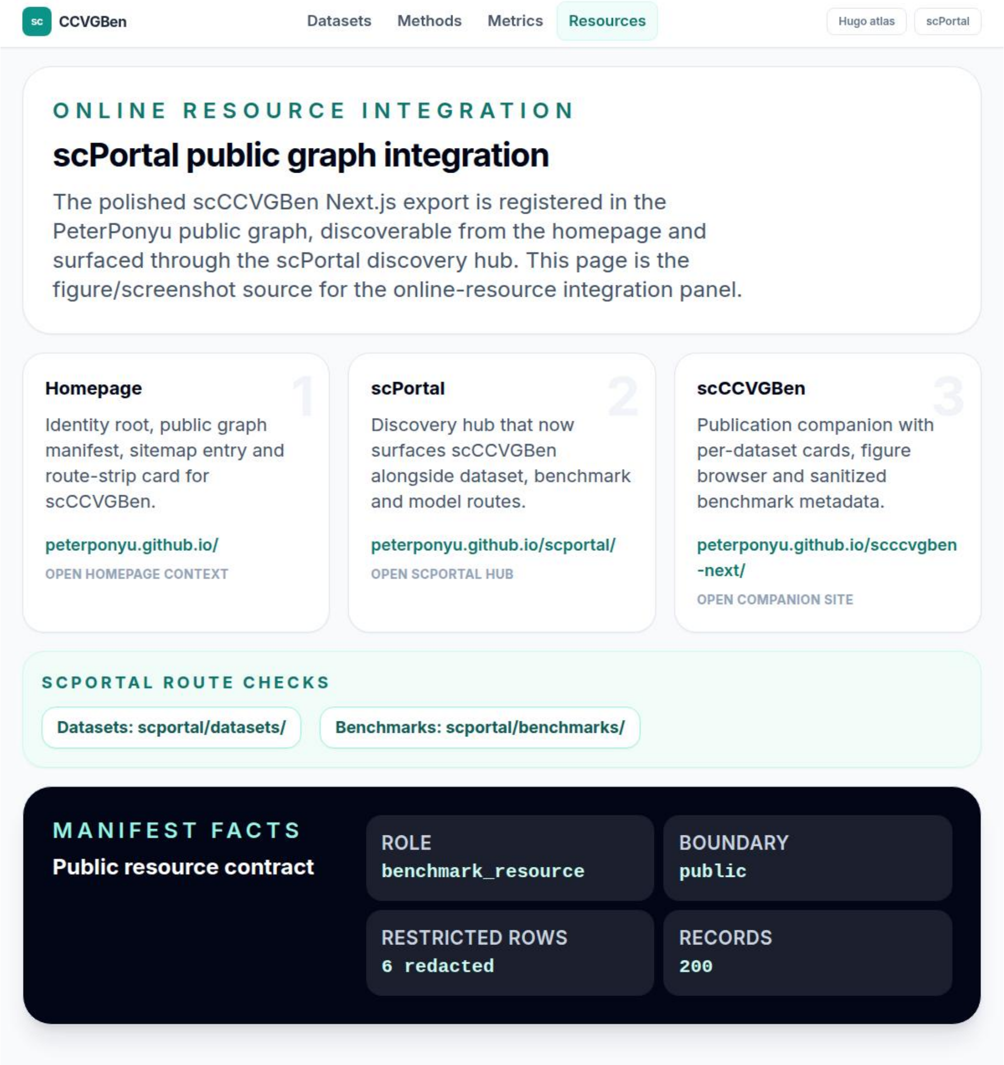

Fig. S2 Online resource integration — scPortal ecosystem layer with public-graph integration. The scPortal site at https://peterponyu.github.io/scportal/ provides the ecosystem layer that surrounds the primary scCCVGBen portal documented in the previous figure; it is a cross-tool discovery surface where the scCCVGBen benchmark cohort and its methods are registered as a public graph alongside related single-cell tooling, so the dataset records reported here can be discovered without cloning the source repository. The relationship is asymmetric: scCCVGBen is the primary portal that owns the benchmark cohort, the figures and the rebuttal-traceable artefacts; scPortal is the ecosystem context that links the cohort and its methods into a broader public graph. The visual content is sourced from the route shown on the scPortal public page and is intended to remain legible when rendered inside the 17 cm × 21 cm manuscript envelope.

Hyperparameter sensitivity (D2)

The default values in Appendix Table A2 were selected on a held-out validation subset and frozen before the cohort-scale benchmark; the evaluation in the main-text cross-method benchmarks on scRNA-seq and scATAC-seq therefore reflects a single fixed configuration rather than per-dataset tuning. To document the local landscape around that fixed configuration, an OAT (one-axis-at-a-time) sweep was launched on the GSE183904_GastricHmCancer dataset varying $\beta \in \{0.5, 1, 2, 4\}$, $\alpha \in \{0.1, 0.5, 1\}$, $w_{\text{adj}} \in \{0, 0.5, 1, 2\}$, dropout $\in \{0, 0.05, 0.2\}$, and hidden dim $\in \{64, 128, 256\}$. The completed sweep cells are released alongside the manuscript with the public source repository (one CSV per axis-value pair) and aggregate to spreads of $\Delta^{\beta}_{\text{ASW}} \approx 0.034$ and $\Delta^{w_{\text{adj}}}_{\text{ASW}} \approx 0.005$ across the released cells. Because the OAT scan is single-dataset by design and is not paired against the cohort-scale benchmark of the main-text scRNA-seq cross-method analysis, we restrict the quantitative claims in the main text to the fixed-configuration cohort results and document the per-axis spreads here as a reproducibility protocol; cohort-scale generalisation of the OAT scan is identified as a future-extension item.

Cluster-algorithm and seed sensitivity (D5, D6)

Two clustering-sensitivity analyses ground the metric-driven decline noted in the main-text scRNA-seq cross-method analysis and the seed-driven stability claim of the main-text centroid-stability analysis. **D5-partial** (cohort scale) recomputes the Spearman rank correlation of method orderings across ARI, NMI, and ASW for every dataset that yields ≥ 3 scoring methods. Across $n = 55$ datasets with well-defined correlations,

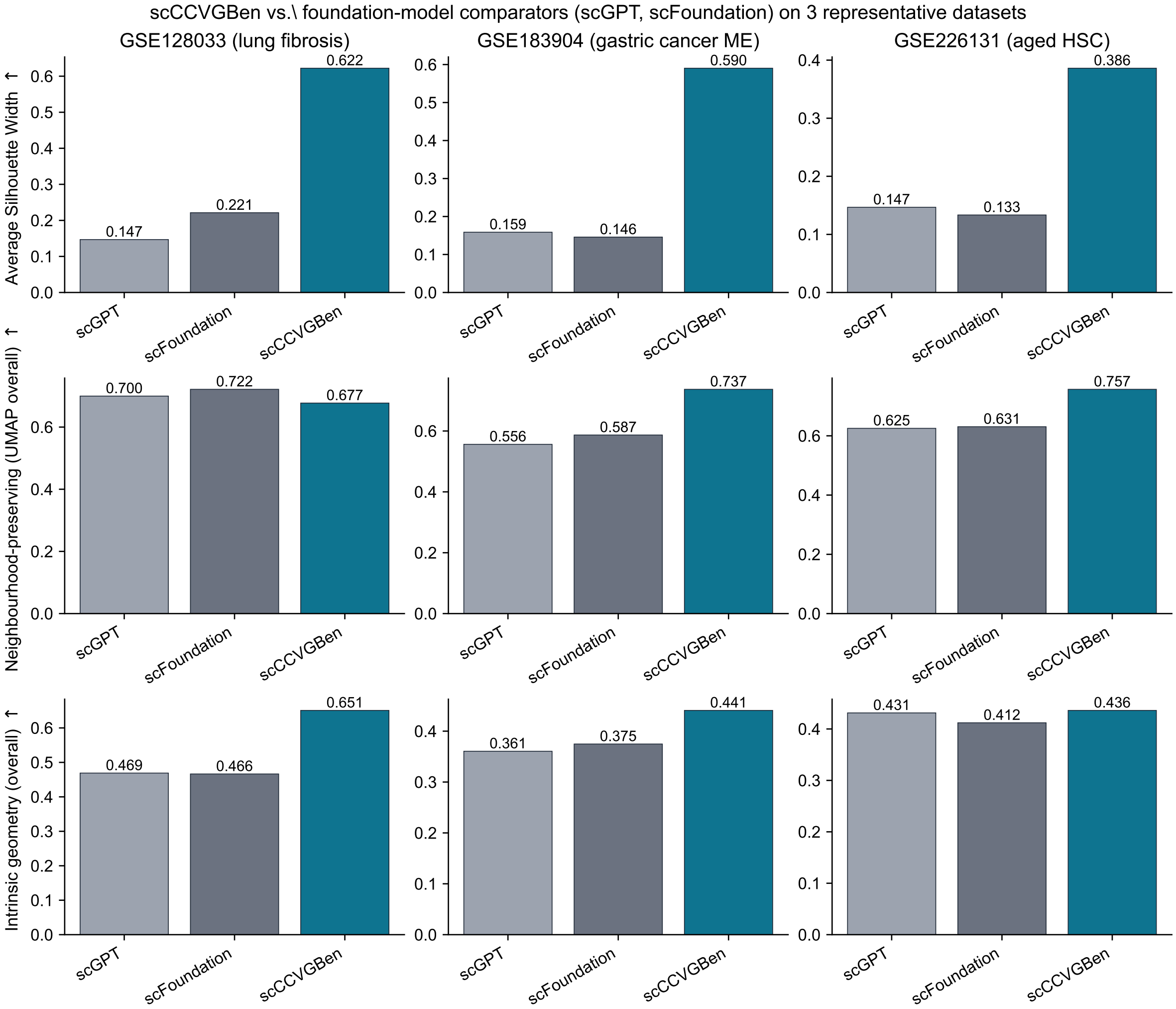

Fig. S3 Foundation-model comparison: scCCVGBen vs. scGPT vs. scFoundation on three reference datasets. The columns report Average Silhouette Width (top row), Overall UMAP neighbourhood preservation (middle row) and Intrinsic geometry overall (bottom row) for scCCVGBen against the published scGPT whole-human checkpoint and the scFoundation 0.1B checkpoint, evaluated within the same per-dataset paired protocol. All nine cells (three datasets × three methods) are populated. Mouse cohort handling: GSE226131 gene symbols are upper-cased to expose orthologs to the human-vocabulary scGPT checkpoint, lifting the vocabulary match from 17 to 15,935 of 31,053 genes; the GSE226131 scFoundation cell required a cold-start inference process to avoid PyTorch allocator fragmentation that accumulates across sequential cohort runs on the 11.7 GB GPU. Across all six reported foundation-model deltas scCCVGBen improves Average Silhouette Width: +0.475 / +0.432 / +0.239 over scGPT and +0.401 / +0.445 / +0.253 over scFoundation on GSE128033 / GSE183904 / GSE226131 respectively, with parallel improvements on intrinsic geometry except for the mouse–scGPT pair which is tied at +0.005. Latents are computed via `scgpt.tasks.embed_data` (whole-human checkpoint, `use_fast_transformer=False`) and via `scfoundation.model.get_embedding` (cell-key, `tgthighres f1`) on the same per-dataset cells used for the scCCVGBen reference. Reconciled foundation-model comparison tables are released with the public source repository.

$\bar{r}_{\mathrm{ARI,NMI}} = 0.996$ (SD 0.019, range 0.900–1.000), $\bar{r}_{\mathrm{ARI,ASW}} = 0.573$ (SD 0.305), and $\bar{r}_{\mathrm{NMI,ASW}} = 0.571$ (SD 0.308); ARI and NMI never disagree on method ordering on this cohort. **D5-full** (latent scale) re-clusters the persisted scCCVGBen latents on three reference datasets across fourteen encoder backbones ($n = 45$ (dataset, method) pairs) under Leiden, Louvain and k-means at matched parameters and computes pairwise ARI between resulting partitions: Leiden–Louvain agreement is $\bar{r}_{\mathrm{ARI}} = 0.78 \pm 0.11$, Leiden–k-means is 0.53 ± 0.14, Louvain–k-means is 0.51 ± 0.13. **D6** (multi-seed Leiden) repeats Leiden five times with seeds $\{0, 1, 2, 3, 4\}$ on the same 45 pairs and reports the median pairwise ARI as 0.811 ± 0.111 (cited inline in the main-text centroid-stability analysis). The cluster-sensitivity, cluster-algorithm and multi-seed CSVs are released with the public source repository and reproduce these numbers from the persisted latents.
